# The soluble G protein of respiratory syncytial virus promotes viral dissemination via TLR2-mediated NLRP3 priming and pyroptosis

**DOI:** 10.1038/s44298-026-00172-x

**Published:** 2026-01-27

**Authors:** Robert Meineke, Ayse Agac, Marie-Christin Knittler, Albert D.M.E. Osterhaus, Martin Ludlow, Guus F. Rimmelzwaan

**Affiliations:** Research Center for Emerging Infections and Zoonoses, University of Veterinary Medicine, Hannover, Germany

**Keywords:** Cell biology, Diseases, Immunology, Microbiology, Pathogenesis

## Abstract

Respiratory syncytial virus (RSV) causes severe lower respiratory tract infections in infants, immunocompromised individuals, and older adults. Although vaccines and monoclonal antibodies have recently become available, understanding RSV pathogenesis remains essential for next-generation therapeutic strategies. RSV attachment glycoprotein G mediates virus binding through a CX3C-like chemokine motif, and its secreted soluble form (sG) possesses immunomodulatory properties. We showed that recombinant sG binds TLR2, inducing proinflammatory mediators. In vitro, sG pretreatment of airway epithelial cells enhanced viral replication upon infection, indicating functions beyond canonical receptor binding. We demonstrated that RSV sG can activate MyD88-NF-κB signalling in uninfected cells via TLR2, leading to NLRP3 upregulation and ROS accumulation. Subsequent RSV infection provides the second signal for caspase‑1 activation and pyroptosis, preconditioning neighbouring cells for inflammasome-dependent lysis and viral egress. Targeting the sG-TLR2 interface could reduce inflammatory damage and viral spread, providing a rationale for CX3C motif-directed interventions and NLRP3 inhibitors during infection.

## Introduction

Respiratory syncytial virus (RSV), a member of the *Pneumoviridae* family, is a major human respiratory pathogen and a leading cause of acute lower respiratory tract infections across all age groups^1^. Most children encounter RSV early in life, with 2–3% requiring hospitalisation for severe complications such as bronchiolitis or pneumonia. Globally, RSV causes ~3.6 million hospitalisations and ~100,000 deaths annually among children under five, with 97% of fatalities in low- and middle-income countries with limited access to supportive care^2–4^. RSV also significantly affects older adults, with hospitalisation rates comparable to those reported during milder influenza seasons (~60%)^5–8^.

The clinical spectrum of RSV spans from mild upper respiratory symptoms to severe lower tract disease marked by airway inflammation, bronchial obstruction, and, in the most severe cases, respiratory failure requiring mechanical ventilation^9^. The pathogenesis of severe RSV disease involves a combination of direct viral cytopathogenicity and harmful inflammatory immune responses^9–11^. RSV infects primarily ciliated airway epithelial cells, leading to epithelial damage, increased mucus production, and impaired mucociliary clearance. The virus induces robust production of proinflammatory cytokines, including interleukin (IL)-6, IL-8, thymic stromal lymphopoietin (TSLP) and IL-33, which promote neutrophil recruitment and type 2 helper T-cell responses^10,12,13^. These inflammatory cascades not only contribute to acute lung injury but also may predispose patients to long-term respiratory complications, including recurrent wheezing and asthma development during childhood^10,12^.

Recently, several prefusion F protein-based RSV vaccines, including RSVPreF3 (GSK, adjuvant), RSVPreF (Pfizer, non-adjuvant) and mRNA-1345 (Moderna), have received regulatory approval. In addition, long-acting F protein-specific monoclonal antibodies (nirsevimab (Sanofi) and clesrovimab (Merck)) have been developed and approved for effective prophylaxis^14–18^. However, major gaps remain in our understanding of RSV pathogenesis and how the virus can cause repeated infections in the presence of maternal antibodies or preexisting immunity^19,20^. RSV has a remarkable capacity to reinfect despite limited antigenic variation. To evade innate immune responses, viruses encode multiple proteins, including the nonstructural proteins NS1 and NS2, which suppress type I interferon production, and the attachment glycoprotein G, which exists in both membrane-bound and secreted forms with distinct immunomodulatory functions^10,21,22^.

The RSV G protein is a heavily glycosylated type II membrane protein comprising an extracellular domain with a central conserved region flanked by two variable mucin-like domains^23^. Within the central conserved region, a CX3C chemokine motif is located, which viruses use to attach to cells that express the fractalkine (CX_3_CL1) receptor CX_3_CR1, including airway epithelial cells and various immune cells^10,24–26^, which facilitates viral entry. Notably, the CX3C motif also displays molecular mimicry of CX_3_CL1^22^; therefore, the G protein can interfere with normal CX_3_CL1-mediated immune cell trafficking and activation^21,24,25,27^.

The secreted soluble form (sG), generated by alternative translation initiation, constitutes ~80% of the total G protein in infected cells^28,29^. Unlike its membrane-bound counterpart, sG functions as a soluble mediator that can act at a distance from infected cells, playing multiple roles in viral pathogenesis. Previous studies have demonstrated that sG functions as a neutralisation decoy, reducing the efficacy of G-specific antibodies by preventing them from binding to virion-associated G proteins^30^. In addition, sG has been shown to modulate inflammatory responses and interfere with Fc receptor-bearing leucocytes, which contribute to antibody-dependent viral clearance^31^. Furthermore, sG was found to inhibit the activation of CX_3_CR1 via its natural ligand CX_3_CL1 and impair monocyte function^21^. The strong conservation of sG across RSV strains suggests that it confers a selective advantage, yet its full spectrum of immunomodulatory functions remains unclear^23,32^.

The innate immune system forms the first line of defence against viral infections, with pattern recognition receptors (PRRs) playing crucial roles in detecting viral components and initiating antiviral responses^33^. Toll-like receptor 2 (TLR2), which often forms a heterodimer with TLR6, recognises various pathogen-associated molecular patterns and activates downstream signalling cascades through the adaptor protein MyD88^34^. Upon ligand engagement, TLR2 signalling leads to the activation of nuclear factor-κB (NF-κB), a master transcriptional regulator that controls the expression of numerous genes involved in innate immunity, inflammation and cell survival^33,34^.

Central to the inflammatory response is the NOD-, LRR- and pyrin domain-containing protein 3 (NLRP3) inflammasome, a multiprotein complex that serves as a critical sensor of cellular stress and danger signals^35–37^. The NLRP3 inflammasome requires two distinct signals for activation: a priming signal that upregulates inflammasome components through NF-κB-mediated transcription and an activation signal that promotes inflammasome assembly and caspase-1 activation^35–39^. The priming signal, typically provided by PRR engagement, leads to the transcriptional upregulation of NLRP3, pro-IL-1β and pro-IL-18. The activation signal can be triggered by various stimuli, including potassium efflux, calcium influx, reactive oxygen species (ROS) generation or lysosomal damage^35–39^.

Upon assembly, the NLRP3 inflammasome recruits and activates caspase-1, which has two critical functions: proteolytic maturation of the proinflammatory cytokines IL-1β and IL-18 and cleavage of gasdermin D (GSDMD)^35,40^. GSDMD cleavage releases its N-terminal pore-forming domain, which oligomerizes in cellular membranes to form large pores that facilitate cytokine release and ultimately lead to pyroptosis, a highly inflammatory form of programmed cell death. Pyroptosis is characterised by cell swelling, membrane permeabilization and the release of intracellular contents, including damage-associated molecular patterns (DAMPs), which can further amplify inflammatory responses^37,40,41^. This form of cell death plays dual roles in viral infections, contributing to pathogen clearance by eliminating infected cells while potentially causing collateral tissue damage and facilitating viral dissemination through cell lysis^38,42^.

Several respiratory viruses, including RSV, have been shown to activate NLRP3 inflammasome signalling^11,38,42–45^. RSV infection activates both signals required for NLRP3 inflammasome activation: NF-κB-mediated priming through TLR2/MyD88 signalling and activation signals provided by viral replication-associated cellular stress^44,46^. However, the spatial and temporal dynamics of inflammasome activation during RSV infection are largely unknown. The observation that lung injury during RSV infection often extends beyond infected tissue suggests that the paracrine action of viral components influences the inflammatory state of neighbouring and more distant cells of the respiratory tract^31,47^. This mechanism could explain the extensive airway inflammation and obstruction in severe RSV cases, independent of the extent of direct viral cytopathology^12^. Although previous studies have shown that RSV activates inflammasome signalling and that the G protein possesses immunomodulatory properties^21,30,44,48,49^, the potential of sG to induce inflammasome activation in uninfected cells has not been investigated. We hypothesised that sG released from RSV-infected cells can prime uninfected cells for increased inflammasome activation upon subsequent viral infection.

In the present study, we tested the hypothesis that RSV sG acts as a TLR2 ligand, as suggested by previous in silico studies^50^, and that it can prime airway epithelial cells for increased NLRP3 inflammasome activation by subsequent infection, leading to an inflammatory cytokine response and increased release of the infection virus via pyroptotic cell death. In this way, sG may contribute to the pathogenesis of RSV infection beyond its role in immune evasion. Our findings provide new insights into RSV pathogenesis and identify potential therapeutic targets for reducing the severity of RSV infection.

## Results

### Secretion of sG from RSV-infected epithelial cells

**Fig. 1:**
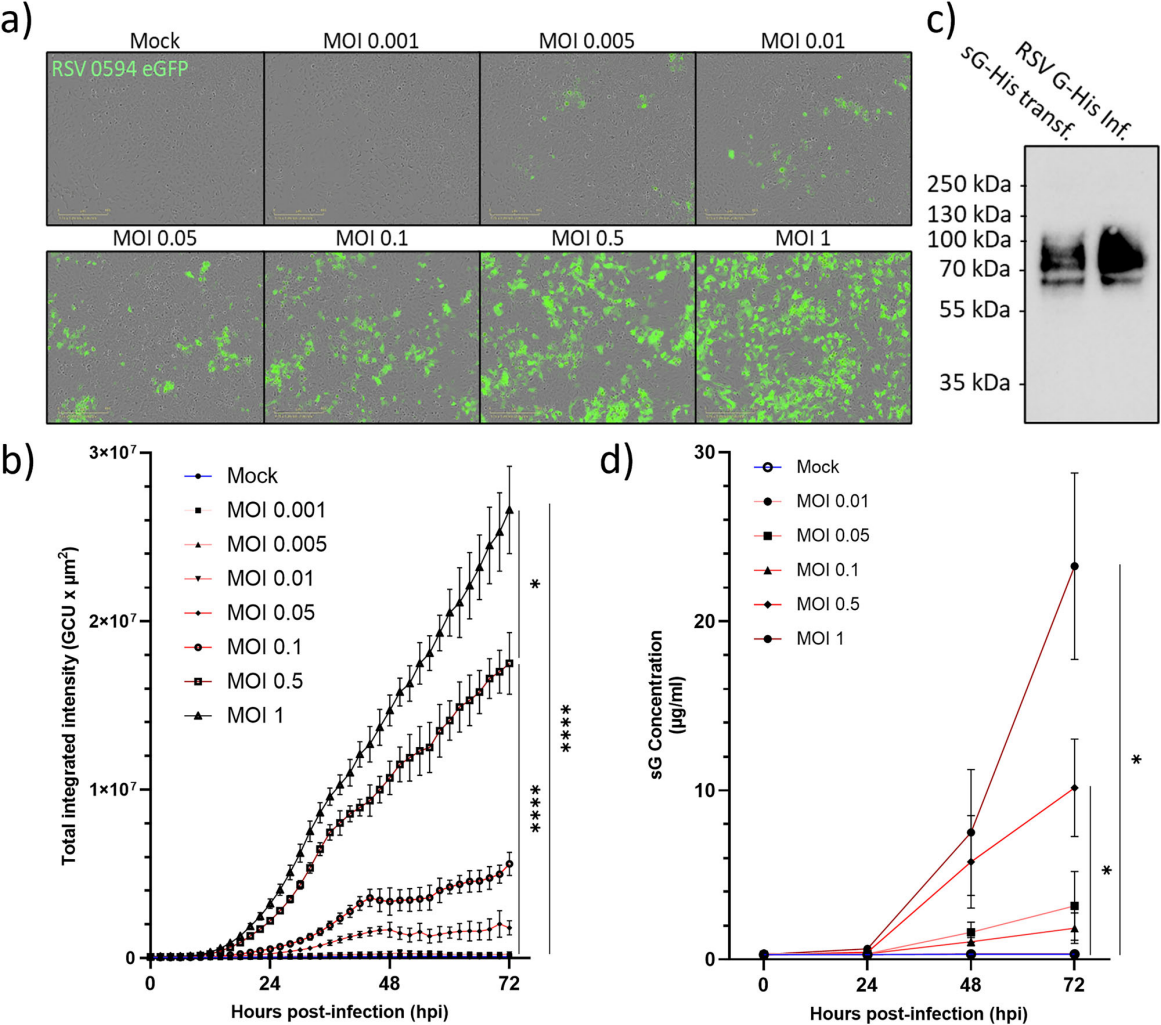
MOI-dependent secretion of sG from RSV-infected epithelial cells. **a** Representative IncuCyte live-cell images of A549 monolayers infected with RSV-A-0594-eGFP at various MOIs (0.001-1) at 72 hpi. **b** Quantitative analysis of IncuCyte live-cell imaging for total integrated GFP intensity normalised to cell coverage. **c** Western blot analysis of His-tagged sG from HEK293T cells transfected with pcDNA3.1-sG-WT or infected with RSV-A-0594-G-6×His via a monoclonal RSV G antibody (Clone 131-2 G). **d** ELISA quantification of secreted sG in A549 cell supernatants via a 4PL standard curve of purified recombinant sG. The data represent the means ± SDs of three independent experiments. Statistical analysis: **c** one-way ANOVA with Tukey’s post hoc test. **d** Kruskal–Wallis test with Dunn’s post hoc test; *P < 0.05, **P < 0.01, ***P < 0.001, ****P < 0.0001.

To assess soluble G protein (sG) secretion kinetics during RSV infection of lung epithelial cells, A549 cells were infected in parallel with RSV-A-0594-eGFP or RSV-0594-G-His with MOIs ranging from 0.001-1 to demonstrate the correlation between de novo protein biosynthesis via eGFP expression and the detection of His-tagged G protein secreted from RSV-0594-G-His infected cells in ELISA. IncuCyte live-cell imaging revealed an MOI-dependent increase in the number of GFP-positive cells at 72 hpi (Fig. 1a). Quantitative analysis of the total integrated GFP intensity normalised to the cell density confirmed the occurrence of MOI-dependent GFP expression over time (Fig. 1b).

After infection of A549 cells with RSV-A-0594-G-His, the culture supernatant was tested for the presence of His-tagged sG by western blotting, which revealed that sG species migrated between 60 and 110 kDa (Fig. 1c). The molecular weight of sG produced after RSV infection resembled that of His-tagged sG purified from HEK293T cells transfected with pcDNA3.1-sG-WT. Finally, the cell culture supernatants of the RSV-A-0594-G-His-infected cells were first passed through 0.1 µm Whatman Anotop syringe filters to remove RSV particles, and the clarified samples were subsequently subjected to ELISA, where sG production, quantified via the use of purified recombinant sG as the standard, was MOI‑dependent and time‑dependent, reaching 23 µg/mL at an MOI of 1 at 72 hpi (Fig. 1d).

### Recombinant sG binds to GAGs, CX_3_CR1 and TLR2

**Fig. 2:**
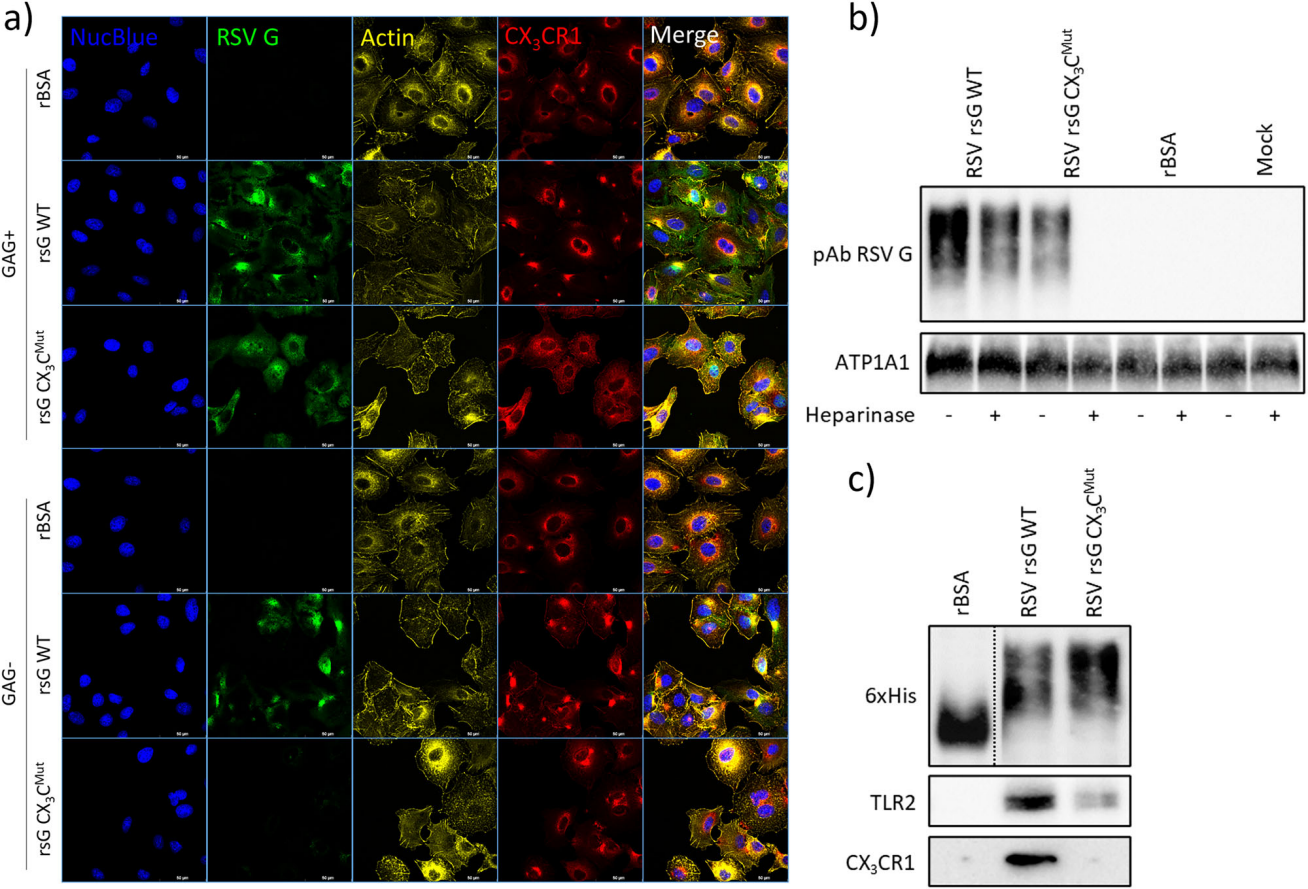
Analysis of sG binding to cellular receptors and membranes. **a** Confocal microscopy of A549 cells treated with recombinant wild-type sG (rsG WT), CX₃C-mutant sG (rsG CX₃Cmut), or rBSA, with or without heparinase I/III digestion. The cells were stained for RSV G (green), CX3CR1 (red), F-actin (yellow) and nuclei (blue). **b** Western blot of membrane fractions probed for RSV G and ATP1A1 as loading controls. **c** His-tag pull-down assay with subsequent Western blot detection of bait protein (via HisTag), TLR2 and CX3CR1.

To investigate the receptor-binding properties of recombinant RSV sG, we combined glycosaminoglycan (GAG) removal, confocal colocalization imaging and His-tag pull-down assays (Fig. 2a–c). Heparinase I/III digestion efficiently stripped GAGs from A549 cells (Supplementary Fig. 1). In order to investigate the dependency of a functional CX3CR1 binding motif on the biological properties of the RSV sG protein, both wildtype sG was used and a mutant with a mutation in the CX3C motif (sG CX3CMut), known to abrogate binding to CX3CR1 (C186R) in this and all subsequent experiments. We applied rBSA alongside the recombinant viral proteins, as a negative control, produced under identical expression and purification conditions as the sG proteins. In contrast to rBSA, both recombinant wild-type sG (sG WT) and the CX_3_C-mutant sG (sG CX_3_C^Mut^) bound to untreated A549 cells. After GAG removal, the sG CX_3_C^Mut^ failed to bind, whereas the sG WT retained punctate binding overlapping with CX_3_CR1 staining. After sG binding, CX_3_CR1 expression displayed a more clustered pattern, suggesting ligand-induced relocalization. Nuclear (NucBlue) and F-actin (ActinRed) staining remained unchanged under all conditions (Fig. 2a).

The binding of RSV sG to A549 cells was confirmed by Western blotting of the membrane fractions of cells that were or were not treated with heparinase. RSV sG WT was detected irrespective of the type of heparinase treatment, but the signal was strongest in untreated cells (Fig. 2b). In contrast, sG CX_3_C^Mut^ was detected in untreated cells but not in Heparinase-treated cells. rBSA served as a negative control and was not detected in any of the membrane fractions. Detection of ATP1A1 confirmed equal loading (Fig. 2b).

Pull-down experiments with Ni^2+^ magnetic beads further confirmed the binding of RSV sG to CX_3_CR1, whereas RSV sG CX_3_C^Mut^ and BSA, which were included as negative controls, failed to pull down CX_3_CR1 (Fig. 2c). Notably, TLR2 was readily detected with RSV sG WT, whereas with sG CX_3_C^Mut,^ only a faint TLR2 band was observed. The elution of all bait proteins from the Ni^2+^-magnetic beads was verified by 6 × His probing. Overall, we showed that RSV sG binds cell-surface GAGs, CX_3_CR1 and TLR2 and that an intact CX3C motif is required for high-affinity CX_3_CR1 binding and robust TLR2 recruitment. Importantly, this pulldown assay was designed not to newly demonstrate sG-CX_3_CR1 binding, but rather to confirm the loss of detectable CX_3_CR1 interaction by the CX_3_C-mutated sG compared to wildtype sG.

### Extracellular sG increases RSV production

**Fig. 3:**
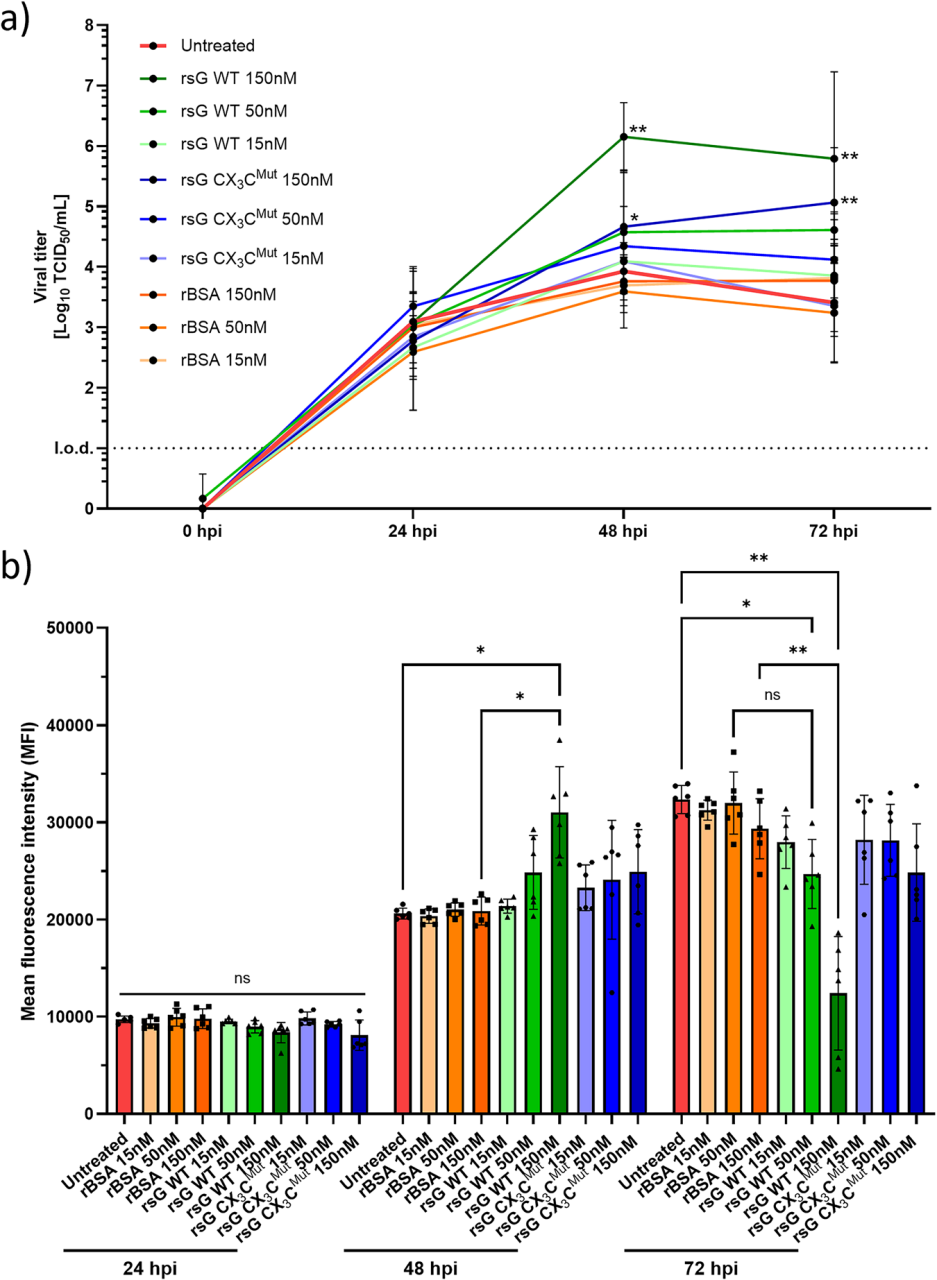
Effect of extracellular sG on RSV replication in A549 cells. **a** TCID₅₀ assay of supernatants from A549 cells infected with RSV-A-0594-eGFP (MOI of 1) and supplemented with recombinant sG (WT or CX_3_Cmut) or rBSA at the indicated concentrations. **b** GFP mean fluorescence intensity (MFI) measured in infected wells over time. The data are shown as the means ± SDs from three independent experiments. Statistical analysis: two-way ANOVA with Tukey’s post hoc test; *P < 0.05, **P < 0.01, ***P < 0.001, ****P < 0.0001.

To determine whether extracellular sG modulates RSV replication in A549 cells, they were infected with RSV-A-0594-eGFP (MOI of 1) in the presence of various concentrations of sG WT, sG CX_3_C^Mut^ or rBSA. Infectious virus titres were measured in culture supernatants at various time points post infection (Fig. 3a).

The untreated cells reached maximal titres of 10^3·9^ TCID₅₀ mL^−1^ at 48 hpi. Supplementation with 150 nM sG WT increased the titre to 10^6.2^ TCID₅₀ mL^−1^ by 48 hpi. The addition of 150 nM sG CX_3_C^Mut^ also increased virus replication, with titres of 10^5.1^ TCID₅₀ mL^−1^ at 72 hpi. Additionally, the addition of 50 nM sG WT resulted in a moderate but significant increase in the titre to 10^4^·^6^ TCID₅₀ mL^−1^ at 48 hpi. With 15 nM RSV, sG WT did not increase virus production. Low doses of sG CX_3_C^Mut^ (<150 nM) or any BSA control did not increase the viral yield.

Monitoring GFP expression in the same cells revealed the kinetics of virus replication early after infection (Fig. 3b). At 48 hpi with RSV-A-0594-eGFP, the mean fluorescence intensity (MFI) in cells treated with 150 nM sG was significantly greater than that in untreated or rBSA-treated controls. At 72 hpi, this pattern reversed. The GFP MFI in cells treated with 150 nm sG WT was significantly lower than that in untreated cells or those treated with rBSA. Collectively, these data indicate that extracellular sG WT enhances infectious RSV production in A549 cells and that a functional CX3C motif contributes to this effect, which coincides with reduced GFP signals at later stages of infection, possibly reflecting increased virus-induced cytopathogenicity.

### RSV sG Activates TLR2-MyD88 and induces the production of proinflammatory cytokines and chemokines

**Fig. 4:**
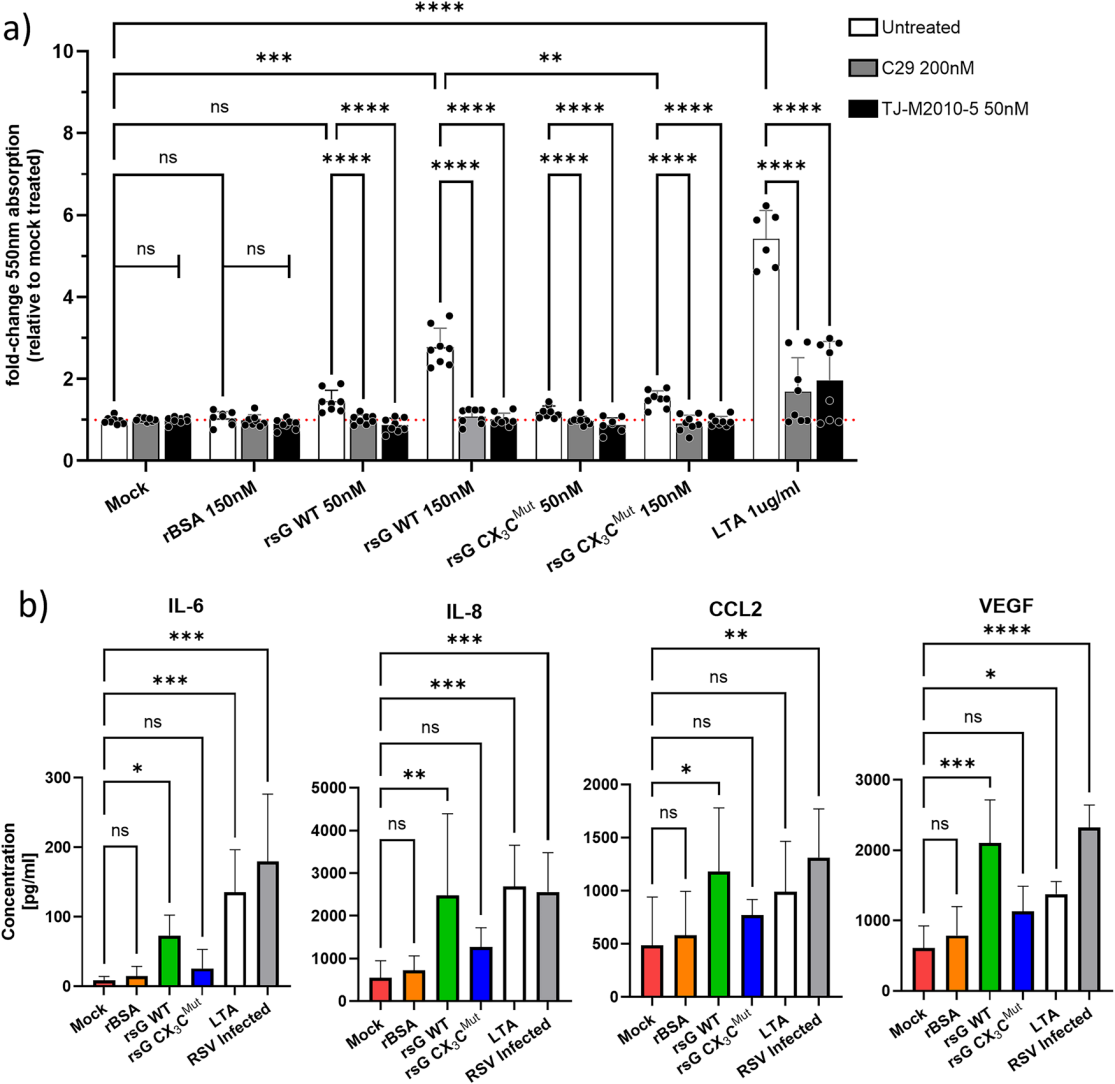
Activation of the TLR2-MyD88 pathway and cytokine release by sG. **a** NF-κB reporter assay in THP-1-Blue cells treated with rsG WT, rsG CX₃Cmut, rBSA, or LTA in the presence or absence of TLR2 or MyD88 inhibitors. SEAP secretion was measured at 550 nm. **b** Luminex multiplex analysis of IL-6, IL-8, VEGF and CCL2 in A549 cell supernatants after treatment. The data represent the means ± SDs from three independent experiments. Statistical analysis: **a** Two-way ANOVA with Tukey’s post hoc test. **b** Kruskal‒Wallis test with Dunn’s post hoc test; *P < 0.05, **P < 0.01, ***P < 0.001, ****P < 0.0001.

Since RSV sG has been shown to interact with TLR2, we next wished to determine whether sG activates TLR2 signalling and induces innate responses. To this end, we used THP-1-Blue NF-κB reporter monocytes, which secrete embryonic alkaline phosphatase (SEAP) when NF-κB is activated downstream of TLR signalling pathways (Fig. 4a).

Stimulation of THP-1-Blue NF-κB reporter cells with 150 nM sG WT induced robust SEAP secretion, which was significantly inhibited by either the TLR2 inhibitor C29 or the MyD88 inhibitor TJ-M2010-5, confirming the activation of the TLR2/MyD88 axis (Fig. 4a). Stimulation with 50 nM sG WT or sG CX_3_C^Mut^ did not increase NF-κB activation compared with mock- or BSA-treated cells, indicating that a functional CX3C motif was essential for activation. The canonical TLR2 agonist lipotheichoic acid (LTA) was used as a positive control and induced robust NF-κB activation that was significantly inhibited by C29 and TJ-M2010-5.

The immunostimulatory properties of RSV sG were further investigated by stimulating A549 cells. (Fig. 4b). Compared with mock controls, WT RSV stimulated robust secretion of IL-6, IL-8, VEGF and CCL2 from A549 cells, as measured by multiplex Luminex assay (Fig. 4b). Sixteen of the other analytes were not induced by sG (data not shown). Stimulation with BSA or sG CX_3_C^Mut^ did not result in the induction of significant cytokine/chemokine secretion. Additionally, infection with RSV-A-0594 induced robust IL-6, IL-8, VEGF and CCL2 production. Similarly, LTA stimulation induced the production of IL-6, IL-8 and VEGF but not CCL2. Collectively, these data demonstrate that RSV sG can activate the TLR2‒MyD88 axis, leading to NF-κB activation and the secretion of proinflammatory cytokines and chemokines.

### RSV sG induces caspase-1 but not caspase-3/7 activity

**Fig. 5:**
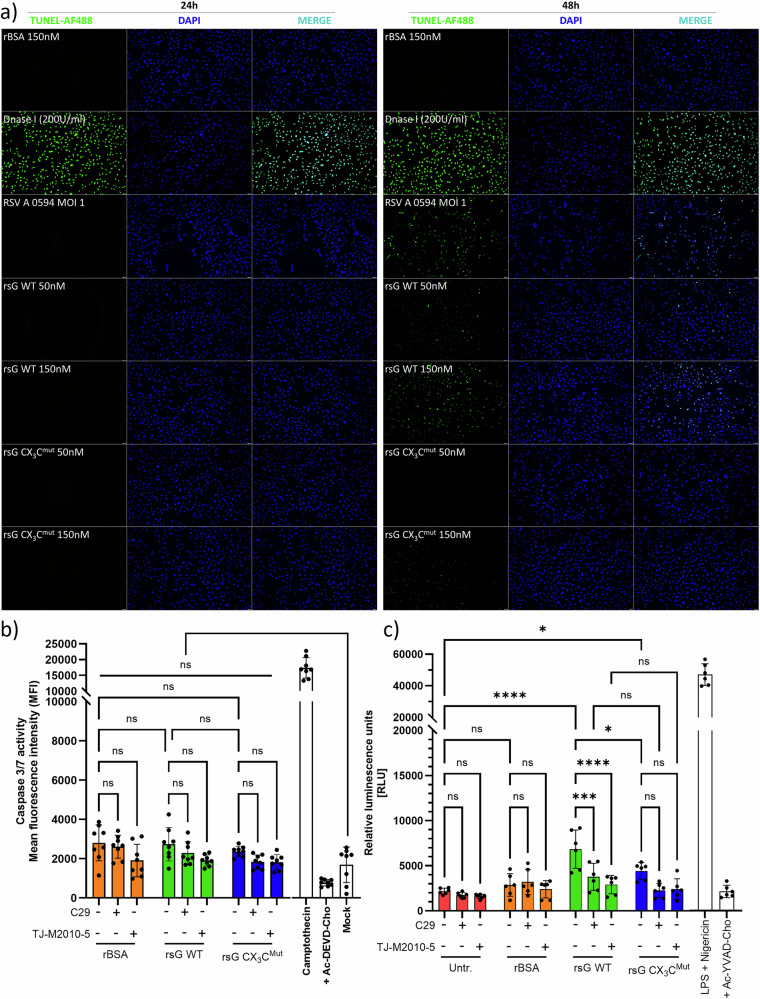
Assessment of apoptotic and pyroptotic cell death following sG treatment. **a** TUNEL assay of A549 cells treated with rsG WT, rsG CX₃Cmut or rBSA or infected with RSV. DNase I-treated cells served as a positive control. **b** Caspase-3/7 activity was measured by a fluorometric assay; camptothecin and Ac-DEVD-CHO were used as controls. **c** Caspase-1 activity was quantified by a luminescence assay with and without TLR2 or MyD88 inhibitors. **a**, **b** Data are presented as the means ± SDs of replicates from three independent experiments. Statistical analysis: two-way ANOVA with Tukey’s post hoc test; *P < 0.05, **P < 0.01, ***P < 0.001, ****P < 0.0001.

To determine whether sG-induced TLR2 signalling induces apoptotic or pyroptotic death in airway epithelial cells, we performed TUNEL microscopy and caspase-3/7 and caspase-1 activity assays (Fig. 5a–c). TUNEL labelling of A549 cells revealed no DNA fragmentation 24 h after exposure to BSA, sG WT or sG CX_3_C^Mut^, whereas positive control DNase I treatment produced strong nuclear fluorescence (Fig. 5a, 24 h).

At 48 h, a faint TUNEL signal was observed after treatment with sG WT, which was dose-dependent and similar to that observed after infection with RSV-A-0594. Treatment with sG CX_3_C^Mut^ or rBSA did not result in a TUNEL signal. DAPI staining revealed comparable nuclear densities across conditions, except for a slight reduction after RSV infection, which was consistent with the cytopathic effects of infection. Caspase-3/7 activity was quantified as a potential source of TUNEL-detected DNA fragmentation. None of the test conditions increased caspase-3/7 activity above baseline, whereas the positive control camptothecin produced the expected robust signal that was abolished by the caspase-3/7 inhibitor Ac-DEVD-CHO, confirming assay specificity (Fig. 5b). Inhibitor addition had no effect, indicating that RSV sG did not induce apoptosis under these conditions. In contrast, both sG WT and sG CX_3_C^Mut^ increased caspase-1 activity compared with that in the untreated and BSA control groups (Fig. 5c). Caspase-1 activity induced by sG WT treatment (6842 RLU) was greater than that induced by sG CX_3_C^Mut^ (4432 RLU), indicating the importance of the CX3C motif. Pretreatment with C29 or TJ-M2010-5 significantly reduced caspase-1 activity in sG WT-treated cells, confirming the contribution of the TLR2/MyD88 signalling pathway. Stimulation with LPS and nigericin was included as a positive control and induced positive caspase 1 activity, which could be inhibited with Ac-YVAD-CHO, confirming the specificity of the assay. Collectively, these data indicate that RSV rsG does not elicit caspase-3/7-mediated apoptosis but induces TLR2-MyD88 signalling and caspase-1 activation, which is largely dependent on the presence of the CX3C motif and promotes low-level DNA damage, which is consistent with the onset of pyroptotic cell death.

### sG primes RSV-infected epithelial cells to enhance NLRP3 pyroptosis

**Fig. 6:**
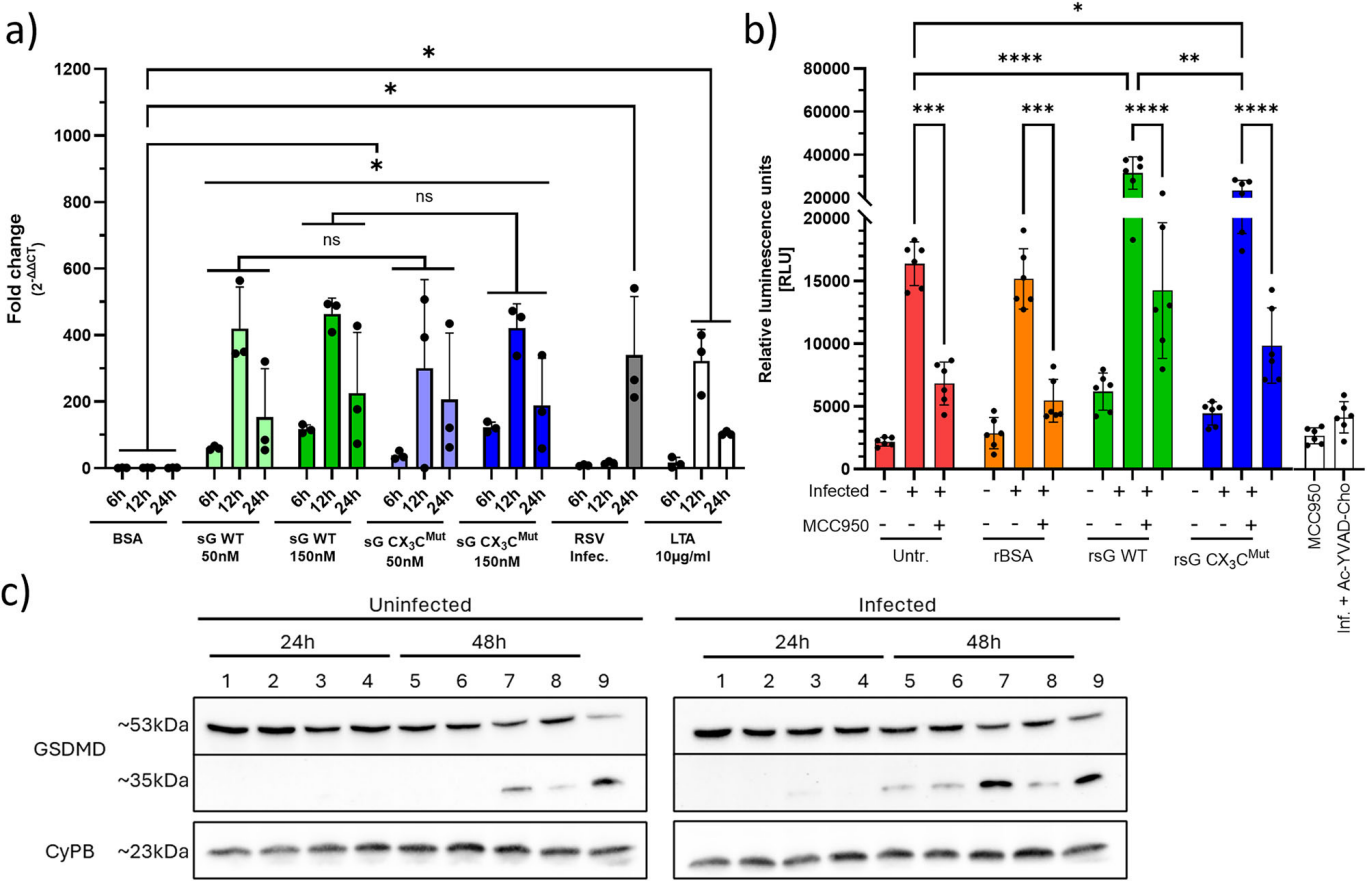
NLRP3 inflammasome priming and activation by sG and RSV. **a** RT‒qPCR analysis of NLRP3 mRNA expression in A549 cells after treatment with sG, rBSA, RSV or LTA at the indicated time points. **b** Caspase-1 activity in A549 cells pretreated with sG and superinfected with RSV, with or without the NLRP3 inhibitor MCC950. **c** Western blot detection of full-length and cleaved gasdermin D (GSDMD) in cell lysates. Lanes 1–4 and 5–8 represent untreated (1/5), rBSA 150 nM (2/6), rsG WT (3/7) and rsG CX3Cmut (4/8). Lane 9 represents in both panels (left (L)/right (R)) a PMA + LPS-treated THP-1 positive control. **a**, **b** Data represent the mean ± SD from three independent experiments. Statistical analysis: two-way ANOVA with Tukey’s post hoc test; *P < 0.05, **P < 0.01, ***P < 0.001, ****P < 0.0001.

Because NLRP3 inflammasome activation requires an NF-κB–driven priming step before assembly and caspase-1 engagement, NLRP3 transcription, caspase-1 activity and gasdermin D (GSDMD) cleavage were assessed after the treatment of A549 cells with sG and subsequent RSV infection (Fig. 6a–c). In order to mimic the in vivo situation, where sG released from RSV-infected cells can stimulate adjacent non-infected cells that subsequently can become infected, A549 cells were pre-treated with sG WT or sG CX_3_C^Mut^ and then infected with RSV. To determine the effect of sG, the outcome of the respective experiments was compared with untreated cells.

Compared with treatment with BSA, treatment of A549 cells with RSV sG WT or sG CX_3_C^Mut^ induced NLRP3 gene expression, as demonstrated by RT‒qPCR, similar to treatment with the TLR2 agonist LTA. NLRP3 mRNA expression peaked at 12 h after treatment (419-, 463- and 323-fold, for sG WT, sG CX_3_C^Mut^ and LTA, respectively) (Fig. 6a). Additionally, after infection with RSV-A-0594, a 339-fold increase in NLRP3 expression was observed at 24 hpi. In contrast, a significant increase in the level of the IL-1β mRNA transcript (Supplementary Fig. 2) was observed only after RSV infection or LTA stimulation but not after stimulation with sG. To test whether priming with RSV sG induced inflammasome activation, caspase‑1 activity was quantified 24 h after treatment in sG‑treated cells with and without subsequent RSV infection, in cells infected with RSV without sG treatment, and in uninfected and untreated controls, each assessed in the presence or absence of the NLRP3 inhibitor MCC950 (Fig. 6b). RSV infection significantly induced caspase-1 activity, which was inhibited by MCC950, confirming that caspase-1 activity was mediated through NLRP3 (Fig. 6b). Priming the cells with sG WT produced a greater caspase-1 response than did priming with sG CX_3_C^Mut^, whereas BSA treatment had no effect. The differences in Caspase 1 activity between sG WT- and sG CX_3_C^Mut^-treated cells were abolished by the addition of MCC950. Finally, GSDMD processing was examined via Western blot analysis (Fig. 6c). In uninfected cells, only faint cleavage products (35 kDa) were detectable at 48 h after treatment with sG WT (Lane 7) or sG CX_3_C^Mut^ (Lane 8); the 53 kDa full-length precursor remained constant. Subsequent RSV infection markedly increased 35 kDa GSDMD-N generation. Cleaved fragments were detectable in untreated, BSA-treated and sG CX_3_C^Mut^-treated cells (Lanes 5, 6 and 8 of the right panel, respectively) and were most pronounced in sG WT-treated cells (Lane 7, right panel), similar to the positive controls PMA and LPS (Lane 9). Collectively, these data show that sG provides a potent priming signal through TLR2 signalling that increases NLRP3 expression, thereby sensitising epithelial cells to NLRP3-dependent caspase-1 activation and GSDMD cleavage upon subsequent RSV infection, which is indicative of pyroptosis.

### RSV sG-mediated and infection-induced NLRP3-dependent loss of metabolic activity

**Fig. 7:**
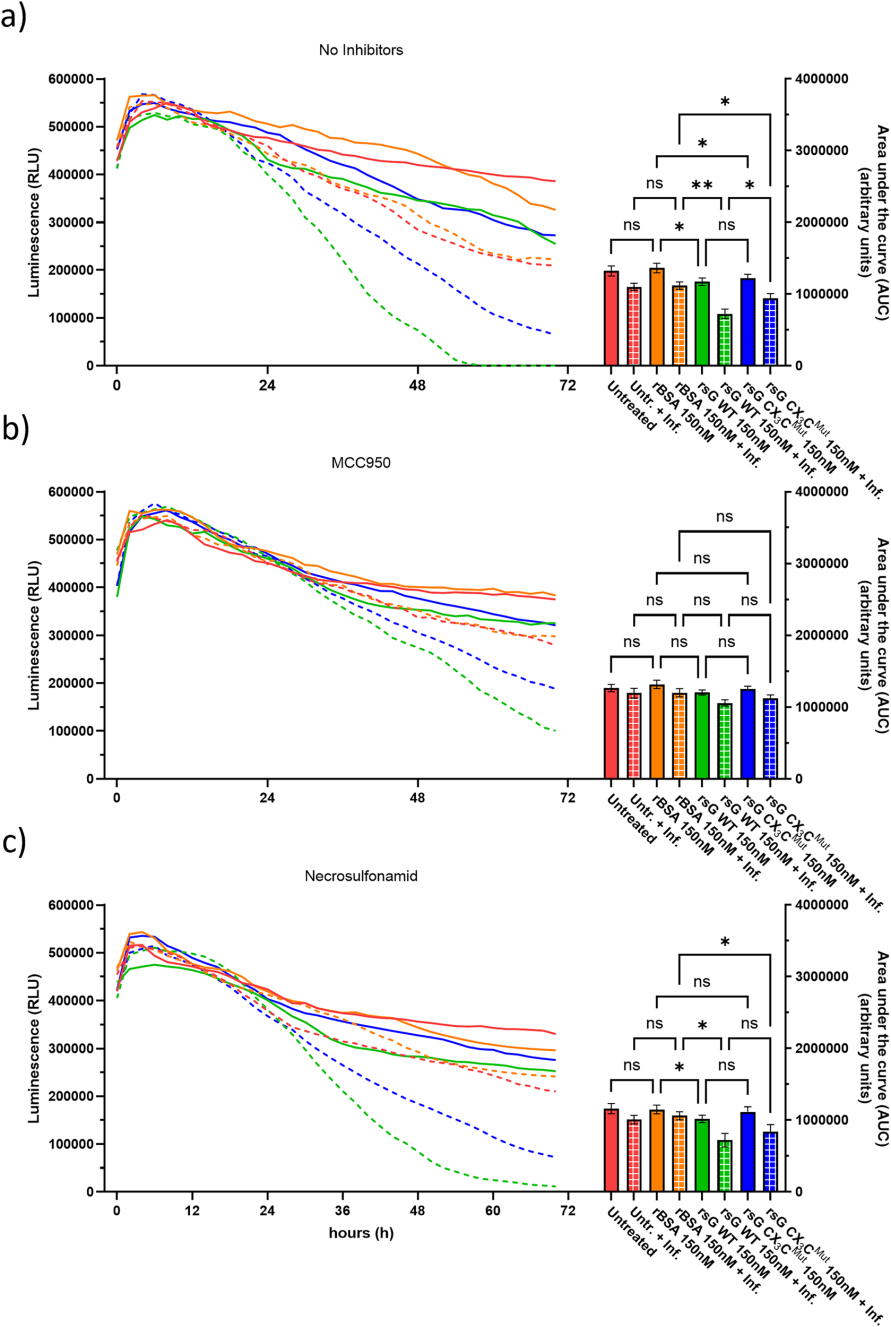
Impact of sG and NLRP3 inhibition on epithelial cell metabolic activity. **a** RealTime-Glo MT cell viability assay in A549 monolayers treated with sG, rBSA or RSV. **b** Effect of the NLRP3 inhibitor MCC950 on metabolic activity. **c** Effect of the gasdermin D inhibitor necrosulfonamide. Luminescence was recorded every 30 min for 72 h. Data are presented as the means ± SDs from three independent experiments. Statistical analysis: area under the curve (AUC) comparison via one-way ANOVA; *P < 0.05, **P < 0.01, ***P < 0.001, ****P < 0.0001.

To further investigate how NLRP3-driven pyroptosis influences overall cell fitness and integrity, we monitored the real-time metabolic activity of A549 cells via the RealTime-Glo MT assay, which detects mitochondrial reductase activity as a sensitive proxy for cell viability (Fig. 7a‒c).

Without infection, sG WT or sG CX_3_C^Mut^ had only modest effects on cell viability compared with the effects of the untreated or BSA controls (Fig. 7a). Additionally, subsequent infection of untreated and BSA-treated cells with RSV reduced cell viability to a limited extent. In contrast, pretreatment, especially with sG WT, profoundly reduced viability upon subsequent infection with RSV. Statistical analysis of the area under the curve revealed that the metabolic activity of RSV-infected cells pretreated with sG WT was significantly lower than that of untreated cells or cells pretreated with sG CX_3_C^Mut^.

To confirm that NLRP3 plays a role in the RSV sG-mediated and infection-induced loss of metabolic activity, experiments were performed in parallel with the continuous presence of the selective inflammasome inhibitor MCC950 (Fig. 7b). MCC950 largely reversed the loss of metabolic activity caused by sG and RSV infection. Differences in the area under the curve were no longer significant between any treatment groups, although a nonsignificant trend toward lower values was detected in the sG WT treatment group/subsequent infection with RSV group, indicating that the sG effect was almost completely compensated for by NLRP3 blockade.

Finally, we tested the effects of necrosulfonamide, an inhibitor of mixed lineage kinase domain-like protein (MLKL), and pore formation by gasdermin-D (Fig. 7c). Necrosulfonamide partially restored the loss of metabolic activity caused by sG and infection but was less effective than the NLRP3 inhibitor MCC950. Differences in the area under the curve between sG WT treatment alone and for both sG variants combined with subsequent RSV infection, compared with their respective BSA controls. Collectively, the results of real-time monitoring of metabolic activity revealed that RSV sG treatment in combination with subsequent RSV infection significantly increased the loss of viability and that this effect was primarily mediated by the NLRP3–gasdermin-D pyroptosis axis.

### RSV sG-TLR2 signalling drives iNOS upregulation and nitric oxide production

**Fig. 8:**
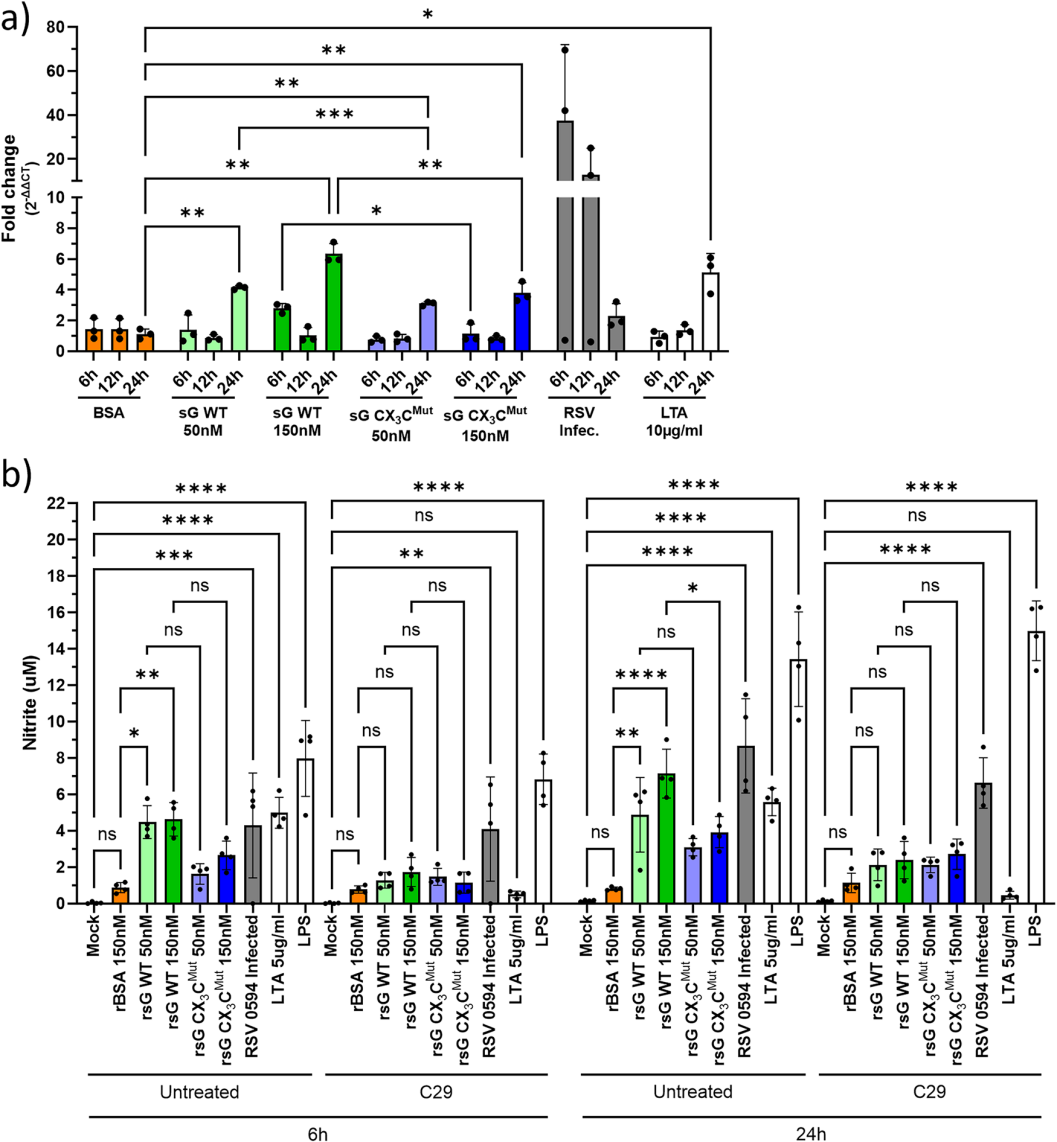
Induction of iNOS and nitric oxide production by sG-TLR2 signalling. **a** RT‒qPCR for iNOS mRNA in A549 cells treated with sG, rBSA, RSV or LTA. **b** Nitrate/nitrite (NOx) levels in supernatants measured by colorimetric assay, with or without the TLR2 inhibitor C29. The data represent the means ± SDs from three independent experiments. Statistical analysis: two-way ANOVA with Tukey’s post hoc test; *P < 0.05, **P < 0.01, ***P < 0.001, ****P < 0.0001.

To determine whether TLR2 activation by RSV sG induces nitric oxide synthase (iNOS) expression, iNOS RT‒qPCR was performed (Fig. 8a). Treatment of A549 cells with BSA, which served as a negative control, did not induce iNOS gene expression. However, treatment with 50 nM or 150 nM sG WT or sG CX_3_C^Mut^ induced significant upregulation at 24 h, similar to the response induced by the TLR2 agonist LTA. RSV infection induced the highest iNOS gene expression levels, which peaked at 6 hpi, suggesting that other activation pathways, such as intracellular RNA sensing, may play a role. We next measured accumulated nitrite/nitrate in culture supernatants via a nitrate-reductase/Griess assay. Treatment with BSA, with or without the TLR2 inhibitor C29, did not result in nitrite production (Fig. 8b). In contrast, RSV sG WT induced dose-dependent nitrite production detectable at 6 and 24 h, and this response was almost completely inhibited by the TLR2 inhibitor C29. Compared with sG WT, treatment with 150 nM sG CX_3_C^Mut^ also increased nitrite concentrations at 24 h, albeit to a lesser extent, and was similarly C29 sensitive. LTA induced nitrite accumulation, which was suppressed by C29, whereas LPS (1 µg/mL) and RSV infection induced robust nitrite production that was not sensitive to the action of C29, which was consistent with the activation of pathways other than TLR2 activation. Collectively, these data demonstrate that TLR2 activation by RSV sG leads to the expression of the iNOS gene and is at least in part dependent on a functional CX3C motif. In contrast, RSV infection activates additional early pathways leading to iNOS gene expression.

### NOX activation by RSV sG generates cytosolic ROS

**Fig. 9:**
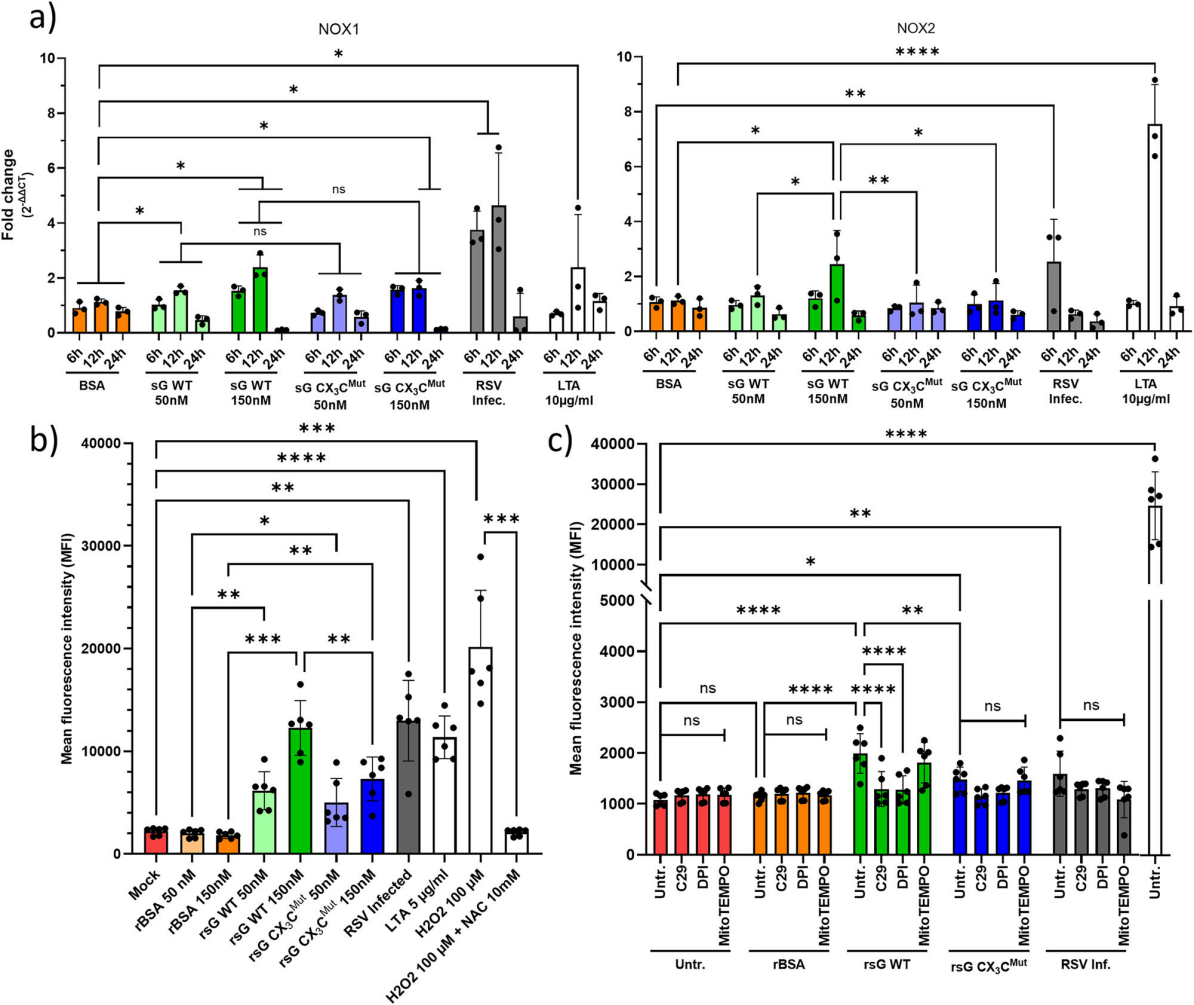
NADPH oxidase activation and ROS/perfoxynitrite generation by sG. **a** RT‒qPCR of NOX1 and NOX2 transcripts in A549 cells treated as indicated. **b** Intracellular ROS levels were measured via the Amplite™ ROS Green assay. **c** Peroxynitrite (ONOO⁻) detection with pathway-specific inhibitors. The data are presented as the means ± SDs from three independent experiments. Statistical analysis: two-way ANOVA with Tukey’s post hoc test; *P < 0.05, **P < 0.01, ***P < 0.001, ****P < 0.0001.

To determine whether RSV sG-induced pyroptosis involves reactive oxygen species (ROS) generation, we first examined NADPH oxidase (NOX) enzyme expression via qPCR (Fig. 9a) and subsequently quantified the intracellular ROS levels (Fig. 9b).

RSV sG WT induced both NOX1 and NOX2 expression in a dose-dependent manner, and the response peaked at 12 h after the start of treatment. For NOX2, the difference between sG WT and sG CX_3_C^Mut^ was statistically significant. The positive control LTA elicited strong responses, especially for NOX2, whereas BSA failed to induce NOX gene expression. Notably, infection with RSV also induced NOX expression, which was already detectable at 6 hpi, suggesting that signalling pathways other than TLR2 signalling were activated by infection.

Next, ROS levels were determined after stimulation with the indicated proteins or after RSV infection. (Fig. 9b). Compared with stimulation with BSA, both sG WT and sG CX_3_C^Mut^ treatment significantly increased ROS levels in a dose-dependent manner. The level of ROS production after treatment with sG WT was significantly greater than that after treatment with sG CX_3_C^Mut^. Compared with 150 nM sG WT, LTA and RSV infection also induced robust ROS production. The positive control hydrogen peroxide (100 µM) produced the strongest signal, which was abolished by N-acetylcysteine (10 mM) pretreatment, confirming the specificity of the assay.

To assess whether the induction of ROS and NO leads to the formation of the potent oxidant peroxynitrite (ONOO^−^), we used a fluorometric ONOO⁻ detection assay and pathway-specific inhibitors (Fig. 9c). ONOO^−^ levels were significantly elevated after stimulation of A549 cells with sG WT, sG CX_3_C^Mut^ or RSV infection compared with those in untreated controls, but only treatment with sG WT reached statistical significance compared with the BSA control (Fig. 9c). Pretreatment with the TLR2 inhibitor C29 or the NOX inhibitor diphenyleneiodonium (DPI) blocked ONOO^-^ formation, whereas MitoTEMPO had no inhibitory effect. As DPI treatment, which targets cytoplasmic ROS generation, blocked the generation of ONOO^-^ while the mitochondrion-targeted antioxidant had no effect, we confirmed the cytoplasmic origin of the ROS by JC-1 mitochondrial membrane potential measurements (Supplementary Fig. 3), which revealed no depolarisation with rBSA, rsG or LTA, unlike the positive control CCCP. The ONOO^-^ levels induced with sG WT (1993 fluorescence units) were relatively low compared with those obtained with the SIN-1 positive control-induced ONOO^-^ levels (24,670 fluorescence units). Considering that other possible signalling pathways can activate pyroptosis, potassium efflux was assessed via the K⁺-sensitive dye PBFI-AM (Supplementary Fig. 4). Treatment with BSA, sG WT, or sG CX_3_C^Mut^ failed to induce detectable K⁺ efflux. In contrast, both RSV infection and the ionophore valinomycin induced significant potassium efflux. Collectively, these data demonstrate that RSV sG induces iNOS and NOX expression through TLR2 signalling, resulting in subsequent ONOO^-^ formation, which could serve as an alternative activation signal for NLRP3 inflammasome activation.

### RSV sG increases NLRP3-dependent LDH release in RSV-infected epithelial cells but fails to induce cytolysis in TLR2^high^ myeloid cells

**Fig. 10:**
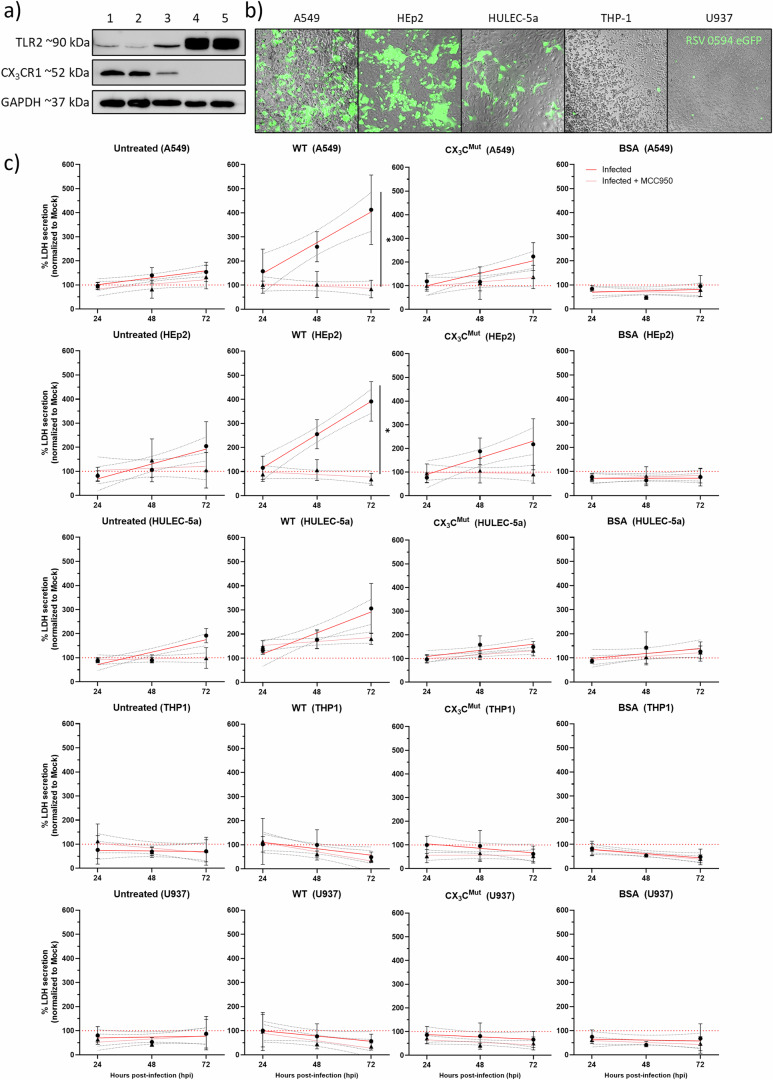
Cell type-specific LDH release following sG and RSV exposure. **a** Western blot analysis of TLR2 and CX3CR1 expression in the indicated cell lines. **b** GFP fluorescence imaging of A549, HEp-2, HULEC-5a, THP-1 and U937 cells after RSV-A-0594-eGFP infection. **c** Quantification of LDH release into the supernatants of RSV-A-0594-infected recombinant protein-treated cells treated with or without the NLRP3 inhibitor MCC950. The data represent the means ± SDs from three independent experiments. Statistical analysis: linear regression and two-way ANOVA with Tukey’s post hoc test; *P < 0.05, **P < 0.01, ***P < 0.001, ****P < 0.0001.

To investigate whether RSV sG has propyroptotic effects in cells other than A549 cells, we used two epithelial lines (A549 and HEp-2), one endothelial cell line (HULEC-5a), and two myeloid lines (THP-1 and U937) differing in CX_3_CR1 and TLR2 expression for comparison (Fig. 10a–c). Western blots confirmed that TLR2 was most abundantly expressed in THP-1 and U937 cells, intermediate in HULEC-5a cells and lowest in A549 and HEp-2 cells. In contrast, CX_3_CR1 expression was opposite to that of TLR2, with the highest expression in epithelial cells and undetectable levels in THP-1 and U937 cells (Fig. 10a). Consistent with CX_3_CR1 expression, 24 h after infection with RSV-A-0594-eGFP, strong GFP fluorescence was observed in A549 and HEp-2 cells, a weaker signal was detected in HULEC-5a cells, and it was detected only sporadically in THP-1 and U937 cells (Fig. 10b). Because lactate dehydrogenase (LDH) is released upon irreversible plasma membrane damage, its accumulation in culture supernatants provides a receptor-independent, broadly applicable readout of lytic cell death for both adherent and suspension cultures. Supernatants collected at 0, 24, 48 and 72 h from cells that were untreated or infected with RSV and treated with 150 nM BSA, sG WT or sG CX_3_C^Mut^ with or without the NLRP3 inhibitor MCC950 were analysed. Linear regression analysis of LDH release over time revealed that RSV sG WT treatment significantly increased cell death in infected A549 and HEp-2 cells, and this effect was reversed by the addition of MCC950. (Fig. 10c). A similar trend was observed in HULEC-5a cells, although this difference was not statistically significant. Treatment of A549 and Hep2 cells with sG CX_3_C^Mut^ increased only cell death to a modest extent, but this effect was not significantly different from that in untreated or BSA-treated cells. In line with their poor CX_3_CR1 expression and poor infectibility, THP-1 and U937 cells presented low MCC950-insensitive LDH levels regardless of sG treatment. Collectively, these findings demonstrate that RSV sG enhances NLRP3-mediated pyroptosis primarily in RSV-permissive epithelial cells, whereas myeloid cells remain refractory despite high TLR2 expression.

## Discussion

In the present study, we quantified the amount of sG released from RSV-infected epithelial cells and identified previously unknown paracrine activities of sG via the use of recombinant protein at equimolar concentrations. We showed that recombinant sG, whose physicochemical properties resemble those of sG released after RSV infection, engages multiple cellular targets, including glycosaminoglycans, the fractalkine receptor CX_3_CR1, and, of special interest, the pattern recognition receptor TLR2. Our findings demonstrate that the interaction of RSV sG with TLR2 initiates a MyD88-NF-κB signalling cascade that drives the production of selected cytokines and provides a priming signal for NLRP3 inflammasome activation. Upon subsequent infection of sG-primed cells with RSV, intracellular sensing and virus-induced cellular stress led to caspase-1 activation, gasdermin-D cleavage, pyroptotic cell death, and increased viral spread.

Previous studies have shown that up to approximately 80% of the total G protein synthesised during RSV infection is secreted rather than virion-associated^28,29^. However, the actual amount of sG secreted by RSV-infected epithelial cells remains elusive. Here, using a genetically modified RSV expressing a His-tagged G protein, we quantified sG secretion by ELISA and determined concentrations of >20 µg/mL (>250 nM). These concentrations mirrored infection rates and provided guidance for physiologically relevant dosing of recombinant sG in subsequent experiments.

The presence of a second translation initiation site at methionine 48, from which sG is synthesised, is highly conserved across RSV strains^31^, indicating that it provides an evolutionary advantage. The secretion of sG from RSV-infected cells may therefore offer the virus a selective advantage, most likely mediated by a variety of known and unknown proviral virus‒host interactions involving sG^21–23,25,30–32,49^. RSV sG engages various molecules on the cell surface, including GAGs, CX_3_CR1 and TLR2. Previous studies have demonstrated that RSV sG can bind CX_3_CR1 via its CX3C motif and interfere with CX_3_CL1-mediated signalling^21,22^. In silico studies also implicated TLR2 binding^50^, which we confirmed experimentally via pulldown assays. Notably, low-affinity binding to GAGs can increase the local concentration of viral proteins, thereby facilitating high-affinity binding to specific receptors. The SARS-CoV-2 spike protein binds to heparan sulphate before engaging with ACE2^51,52^. Similarly, the binding of monomeric RSV sG is dependent on GAG interactions^53^. Our data confirmed that RSV sG can indeed bind to GAGs, as sG CX_3_C^Mut^, which cannot bind CX_3_CR1, still bound to GAG-positive cells. However, wild-type RSV sG bound to heparinase I/III-treated cells devoid of GAG and could bind CX_3_CR1 directly, as demonstrated by the pull-down assay. Consistent with prior work showing that soluble G can act as a competitive antagonist at CX3CR1, the paracrine effects characterised here are better explained by TLR2 engagement and downstream MyD88–NF-κB priming of NLRP3, rather than by productive activation of CX3CR1 signalling in epithelial cells.

Since RSV sG can engage multiple host receptors, including GAGs, CX_3_CR1 and TLR2, the logical next question is how these interactions translate into effects on viral replication and dissemination. Our data revealed that the treatment of RSV-infected cells with sG significantly increased the viral yield in the culture supernatants, with wild-type sG increasing the titre by up to 2.2 log₁₀ TCID₅₀. Given that RSV is generally considered a cell-associated virus whose spread occurs primarily through syncytium formation^54^, these increases indicate that sG promotes more efficient viral dissemination. Consistent with this, the stronger GFP fluorescence observed at 48 hpi likely reflects an amplification of viral spread driven by the paracrine effects of sG. In infected cells, sG may accelerate virion release (e.g. through enhanced egress from pyroptotic cells), whereas in uninfected cells, it may compromise epithelial integrity or render neighbours more susceptible to infection^55,56^. Importantly, our temporal analysis revealed that both sG-treated and untreated cultures reached peak titres at 48 hpi, indicating that sG amplifies the magnitude of spread without altering RSV replication kinetics. This finding is consistent with sG’s established role as a pathogenesis modifier rather than as a replication enhancer^57–59^. The subsequent decline in GFP signals at 72 hpi (Fig. 3B), along with high extracellular viral titres, suggests that elimination of infected cells by pyroptosis or other death pathways may contribute to sustained virion release, suggesting that epithelial sloughing and prolonged viral shedding are observed in severe RSV bronchiolitis^60,61^.

Previously, we demonstrated that RSV sG can block CX_3_CL1–CX_3_CR1 signalling^21^. Because sG was also found to bind TLR2, we further explored other paracrine biological activities related to downstream signalling of the TLR2 receptor. First, we demonstrated that treatment of an NF-kB reporter cell line led to the activation of these cells, which could be inhibited with the specific TLR2 inhibitor C29 and the MYD88 inhibitor TJ-M2010-5. These data show that the binding of RSV sG to TLR2 leads to its activation and subsequently to the production of cytokines that are under the transcriptional control of NF-kB, including IL-6, IL-8, VEGF and CCL2. In addition, RSV sG induced TLR2-dependent caspase-1 activity, a hallmark of pyroptosis.

Although sG treatment alone induced only modest caspase-1 activity and GSDMD cleavage, it strongly primed cells for increased NLRP3 activation and pyroptosis upon subsequent RSV infection (Fig. 6). This priming effect could be inhibited with the NLRP3 inhibitor MCC950. Respiratory viruses provide both priming and activation signals for NLRP3 inflammasome activation within infected cells^38,42^. Conventionally, K⁺ efflux and ROS formation are the primary activation signals that drive RSV-induced NLRP3 activation^44,62^. In our model, extracellular sG delivers the priming signal via TLR2-MyD88-NF-κB-NLRP3 induction, whereas RSV infection provides the intracellular activation signal. Thus, RSV-infected cells condition their environment by releasing sG, which sensitises surrounding uninfected epithelial cells to subsequent infection. Notably, such priming did not induce high expression of IL-1β in epithelial cells, which is consistent with their known low IL-1β capacity compared with that of macrophages^63^. Our findings agree with those of in vivo studies in TLR2^−/−^ mice, which showed reduced pulmonary inflammation despite unchanged viral loads^44^, findings that can now be explained by the loss of sG-driven priming of NLRP3.

The expression of iNOS and the subsequent production of NO allow TLR2-dependent ONOO^-^ formation through cytoplasmic ROS‒NO interactions, creating a potent oxidising species and additional trigger of inflammasome assembly^64–66^. This provides oxidative stress, in addition to classical reactive oxygen species. The dependence on cytoplasmic rather than mitochondrial ROS sources, confirmed through inhibitor studies and JC-1 membrane potential measurements, suggests a specific NOX1/2-mediated mechanism. These findings are in line with previous reports on the activation of NOX2 in RSV-infected lung epithelial cells^67,68^. The ability of RSV to trigger both classical K⁺ efflux through SH protein viroporin activity^62^ and ROS-NO-dependent ONOO^−^-mediated activation through sG-TLR2 signalling represents a redundant system ensuring robust inflammasome activation across diverse cellular contexts.

The induction of RSV sG-induced pyroptosis was highly cell-type dependent. Epithelial cells (A549, HEp-2) readily underwent pyroptosis, whereas myeloid cells (THP-1, U937) remained refractory despite high TLR2 expression. This selectivity may reflect the inverse expression balance of TLR2 and CX_3_CR1 across cell types. While significant differences in NF-κB activation levels were detected, this did not translate into differential upregulation of NLRP3, suggesting a low threshold for even priming. Additionally, differential CX_3_CR1 expression reflects cell tropism and infectability, providing a secondary activation signal^24,59,69,70^. Thus, epithelial cells, which have high CX_3_CR1 expression but low TLR2 expression, are particularly susceptible to RSV sG-mediated pyroptosis, whereas myeloid cells, which have high TLR2 expression but low CX_3_CR1 expression, are largely refractory. Endothelial cells (HULEC-5a) exhibit an intermediate phenotype and pyroptosis to a limited extent. These findings highlight how RSV exploits receptor patterns to tailor host cell death responses. Comparable strategies are used by other respiratory viruses, such as influenza NS1 (apoptosis modulation)^71,72^ and SARS-CoV-2 ORF3a (pyroptosis control)^73,74^. In contrast to such largely cell-intrinsic mechanisms, RSV sG acts in a paracrine fashion on uninfected neighbours, supporting a model of ‘death-assisted’ viral spread that may not be unique to RSV but may also be employed by other viruses^75,76^.

The elucidation of RSV-sG-activated proviral and proinflammatory pathways identified novel targets for therapeutic intervention^77^. Preventing sG binding via the CX3C motif or blocking TLR2 engagement are promising approaches. Neutralising antibodies targeting the conserved domain of sG^57,78–82^, including high-affinity antibodies such as TRL3D3, which remain effective even at high sG concentrations, have already shown preclinical efficacy^83,84^. Other steps in the RSV sG-pyroptosis pathway could be therapeutic targets for small inhibitory molecules. TLR2 antagonists such as C29, NLRP3 inhibitors such as MCC950 analogues^85^ or gasdermin-D pore blockers represent possible therapeutic intervention approaches with various risk‒benefit profiles. Systemic inhibition of TLR2 signalling may impair innate immunity and cause adverse side effects. Targeting NLRP3 directly may be a more suitable approach, as we have shown that the NLRP3 inhibitor MCC950 protected against RSV sG-mediated cytotoxicity (Fig. 7b), whereas necrosulfonamide offered only partial protection (Fig. 7c). These findings suggest that upstream intervention at the NLRP3 level may be most effective. While necroptosis and pyroptosis have both been described in RSV infections^11,56^ and share upstream triggers, NLRP3 involvement, DAMP release and occur with low caspase 3 activity, the modest impact of necrosulfonamide despite its dual MLKL and GSDMD inhibition argues against a dominant necroptotic role and favours upstream intervention at NLRP3 to mitigate sG-driven cytotoxicity under the conditions tested. Our findings are in concordance with those of previous studies that showed that MCC950 treatment of RSV-infected mice protected against RSV-induced lung immunopathology^39^. Additionally, recently developed vaccines and antibody-based therapies target the RSV F protein^14–18^ and do not consider the role of sG in the pathogenesis of vaccine breakthrough infections. Targeting both F and G proteins may provide broader protection^57,86,87^, as the two proteins fulfil distinct roles: antibodies to F block fusion, whereas antibodies to G neutralise receptor binding and immune evasion, suggesting synergistic benefits of dual targeting.

Although our study provides novel insights into the role of RSV sG in pathogenesis, several aspects warrant further investigation. Most of these experiments were performed in immortalised cell lines infected with RSV at high MOIs, which do not fully recapitulate the natural infection dynamics in primary tissues. Validation in more physiologically relevant airway models, including primary human airway epithelial cells differentiated at air–liquid interface and organoid or ex vivo systems, and ultimately in vivo will be essential to confirm the generalisability of our findings. However, the quantification of sG concentrations in infected cultures indicated that the levels used in our mechanistic studies are physiologically achievable. It is even conceivable that in vivo sG concentrations exceed those observed in vitro. Additionally, the wider applicability of our findings needs to be extended to strains other than the RSV-A ON1 isolate that we used. Lab-adapted strains, such as A2 and type B strains, may exhibit different TLR2-activating or pyroptosis-inducing capacities. The sequence diversity in the G protein variable regions, despite conservation of the central domain, suggests that some strain-dependent differences in sG function may exist. Finally, our findings emphasise the central role of the CX3C motif in mediating sG-dependent effects. The reduced biological activity observed with the CX_3_C-mutant sG strongly supports the functional importance of this motif, although the precise mechanism remains to be fully resolved. This reduced activity could result from structural changes induced by the C186R mutation, which may compromise signalling capacity^88^, or from impaired receptor engagement, which diminishes downstream signalling^59,89^. In support of the latter, we observed that wild-type sG induces CX_3_CR1 clustering (Fig. 2a), which is consistent with receptor-mediated effects^90,91^. Rather than being limited, this mechanistic complexity reinforces the importance of the CX3C motif while highlighting the need for additional structural and biophysical studies, such as surface plasmon resonance^92^ and single-molecule imaging^91^. Thus, while our current work establishes the necessity of this motif for full sG function, a more detailed mechanistic understanding remains an important and promising avenue for future research.

The results of the present study advance our understanding of the role that sG can play in the pathogenesis of RSV infections. In a paracrine fashion, sG can function as a ligand of TLR2 and can prime cells for increased inflammasome activation upon subsequent RSV infection. In this way, sG exerts pro-viral and proinflammatory effects and may be responsible for the extensive inflammation and tissue damage observed after RSV infection, which often exceeds what can be predicted from direct viral cytopathology alone. The identification of RSV sG-induced TLR2 activation and subsequent downstream activation pathways of pyroptosis may aid in the development of specific intervention strategies, which could inhibit viral dissemination, inflammation and tissue damage in severe RSV cases.

## Methods

### Viruses

All infections were performed with the RSV-A-0594 strain (ON1 genotype, 24 amino acid G protein insertion). The 0594 reverse genetics system allows for targeted genomic modifications and subsequent rescue of infectious viruses^93^. Three viruses were used: the parental RSV-A-0594 strain (RSV-A-0594) and recombinant RSV-A-0594 harbouring an eGFP gene between the viral P and M genes (RSV-A-0594-eGFP) as described before^93^. Additionally, a recombinant virus in which a G protein was fused to a 6xHis-tag (RSV-A-0594-G-His) has been generated for this study. Virus stocks were generated by infecting HEp-2 cells at 60–80% confluency in Opti-MEM (Thermo Fisher Scientific) supplemented with 100 IU/mL penicillin and 100 µg/mL streptomycin. Infected cells were incubated at 37 °C and 5% CO₂ until cytopathic effects were evident (3–5 days). Virus-containing supernatants were harvested by scraping, clarified by centrifugation (1000 × *g*, 10 min, 4 °C), precipitated with 10% PEG 6000 (Carl Roth), and centrifuged again (3000 × *g*, 30 min, 4 °C). The pellets were subsequently resuspended in Halt’s balanced salt solution (Gibco) with 20% sucrose, aliquoted, snap-frozen in liquid nitrogen, and stored at −150 °C. Viral titres were determined in HEp-2 cells according to Reed and Muench and expressed as TCID₅₀ per mL.

#### Cell lines

All the cell lines were maintained at 37 °C and 5% CO₂ in media supplemented with 10% FBS (Gibco), 100 IU/mL penicillin, 100 µg/mL streptomycin, and 2 mM L-glutamine unless otherwise specified. 293 T (CRL-3216, ATCC) cells were cultured in DMEM (Capricorn Scientific), and the medium was changed every 2–3 days. The cells were passaged at 70–80% confluency with 0.05% trypsin-EDTA (Gibco). A549 (CCL-185, ATCC) cells were cultured in Ham’s F-12 medium (Capricorn Scientific), with the medium changed every 2–3 days, and passaged at 70–80% confluency with 0.25% trypsin-EDTA. HEp-2 (CCL-23, ATCC) cells were maintained in EMEM and passaged at 80–90% confluency using 0.05% trypsin-EDTA. THP-1 (TIB-202, ATCC) cells were cultured in RPMI-1640 (ATCC 30-2001) supplemented with 0.05 mM 2-mercaptoethanol and subcultured when the density reached 1 × 10^6^ cells/mL. HULEC-5a (CRL-3244, ATCC) cells were maintained in MCDB131 (PAN Biotech) supplemented with 10 ng/mL EGF and 1 µg/mL hydrocortisone. U937 (CRL-1593.2, ATCC) cells were cultured in RPMI-1640 and subcultured at 1 × 10^6^ cells/mL. THP-1-Blue™ NF-κB (InvivoGen) cells were grown in RPMI-1640 with 25 mM HEPES, 100 μg/mL Normocin™, and 10 µg/ml blasticidin. All the cell lines were authenticated via STR genotyping (Microsynth).

#### Viral infections

For all the experiments, the cells were seeded in the appropriate culture format and infected with the indicated multiplicity of infection (MOI) of the corresponding RSV-A-0594 variant in OptiMEM (Thermo Fisher Scientific). The inoculation volume was set at half the standard culture volume for each format to ensure efficient viral adsorption. To minimise the cytotoxic effects of residual polyethylene glycol (PEG) in the virus preparation, all the virus stocks were diluted at least 1:10 in OptiMEM prior to infection. The cells were incubated with the virus for 2 h at 37 °C and 5% CO₂. Following incubation, the inoculum was removed, and the cells were washed twice with prewarmed phosphate-buffered saline (PBS) to eliminate unbound virus and residual PEG. Fresh, prewarmed culture medium—matched the requirements of each cell line and experimental protocol—was then added to each well. The cells were subsequently maintained under standard culture conditions for the duration of the experiment.

#### Plasmids and constructs

All plasmid constructs were generated as described before^21^. The soluble RSV-A G protein (sG) coding sequence (starting from M48) and a CX_3_C motif-deficient variant (C186R—disrupts the CX3C motif by replacing the second cysteine residue with an arginine) were cloned and inserted into pcDNA3.1(+)-C-6His. The empty vector and BSA-6xHis constructs served as controls.

#### Transient transfection of HEK293T cells

HEK293T cells were transiently transfected via the calcium phosphate method as previously described^21^. The cells were seeded at 1 × 10^7^ per 15 cm dish and transfected with 25 µg of plasmid DNA, and the supernatants were harvested 72 h posttransfection, clarified by centrifugation, and used for downstream applications.

#### HPLC protein purification

Proteins were purified via Ni^2+^-affinity chromatography via an ÄKTA Start system with HisTrap Excel columns (Cytiva). Briefly, supernatants of RSV-A-0594-G-6xhis infected or transiently transfected cells were filtered through 0.45 µm cellulose acetate (CA) membrane filters, loaded onto equilibrated HisTrap Excel columns, washed with 10 mM imidazole, and eluted with 500 mM imidazole. The eluates were dialysed against PBS (12-14 MWCO; Merck) and sterile-filtered (0.22 µm), and the endotoxin content was confirmed to be <2.5 EU/mL.

#### Protein quantification and western blotting

Protein concentrations were determined via the Quick Start™ Bradford Assay (Bio-Rad). Defined amounts of protein samples were resolved by 10% SDS‒PAGE and transferred to Immobilon®-P^SQ^ PVDF Membranes (Merck) via the Trans-Blot® Turbo™ Transfer System (Bio‒Rad). The membranes were blocked in 5% skim milk (Carl Roth) in TBS-T (0.1% Tween-20) for 1 h at room temperature, incubated overnight at 4 °C with primary antibodies (see Table 1), washed with TBS-T, and incubated with HRP-conjugated goat anti-mouse IgG (G-21234, Thermo Fisher) and goat anti-rabbit IgG (A27036, Thermo Fisher) secondary antibodies, both at 1:5000, for 1 h at room temperature. Detection was performed using SuperSignal™ West Pico PLUS substrate (Thermo Fisher) and imaged on a ChemiDoc™ system (Bio-Rad).

**Table 1:** Primary antibodies used for western blotting

Target	Manufacturer	Clone/Cat#	Dilution
mAb RSV G glycoprotein	Merck Millipore	131-2G/MM_NF-MAB858-2-5	1:1000
pAb RSV G glycoprotein	Sino Biological	40041-T62	1:1000
6×His tag	Thermo Fisher	HIS-H8/MA1-21315	1:1000
TLR2	Thermo Fisher	JM22-41/MA5-32787	1:500
CX_3_CR1	Thermo Fisher	1H14L7/702321	1:500
Sodium-Potassium ATPase	Thermo Fisher	ST0533/MA5-32184	1:50,000
GSDMD	Thermo Fisher	PD00-18/MA5-44666	1:1000
Cyclophilin B	Thermo Fisher	PA1-027A	1:1000
GAPDH (HRP)	BioLegend	16073	1:10,000

#### Live-cell imaging and quantification

A549 cells were seeded in black, clear-bottom 96-well plates and infected with RSV-A-0594 at MOIs of 0.001-1 or mock-infected. The plates were incubated at 37 °C/5% CO_2_ in the Sartorius IncuCyte S3 system and were imaged every 2 h for 72 h via phase-contrast and GFP channels. Images were analysed for total integrated GFP intensity (GCU × µm^2^) via the cell-to-cell functionality of the IncuCyte S3 software 2021C.

#### Quantification of secreted RSV G protein by ELISA

A549 cells were infected with RSV-A-0594-G-6xHis (MOIs of 0.01-1) or mock-infected in six-well plates. The supernatants were collected at 0, 24, 48 and 72 h, filtered to remove viral particles via Whatman® Anotop® 0.1 μm syringe filters, and stored at −80 °C. Nunc MaxiSorb ELISA plates (Thermo Fisher) were coated overnight at 4 °C with 1 µg/mL mouse anti-RSV G (131-2 G; Merck) in carbonate buffer (Thermo Fisher CNB0011), washed with kit wash buffer, and blocked with kit assay buffer. Samples or defined concentrations of purified recombinant sG protein, which served as a standard, were added, followed by the addition of mouse anti-6 × His (HIS-H8; Thermo Fisher, 1:50,000), and then development with a TMB kit and stop solution. The absorbance was read at 450 nm. Standard curves were generated via a 4PL fit (AssayFit Pro ELISA calculator), and concentrations were normalised to 10^6^ cells/mL.

#### Heparinase I/III digestion of A549 cells

A549 cells were seeded in 48-well plates and digested with 2 U/mL Bacteroides heparinase I (P0735) and III (P0737; NEB) in DPBS + 0.1% BSA for 2 h at 37 °C. Digestion efficacy was confirmed by immunofluorescence staining for HSPGs via an anti-heparan sulphate antibody (clone F58-10E4; AMSBio), followed by Alexa Fluor 594–conjugated goat anti-mouse antibody (0.2 µg/mL; Thermo Fisher), with ActinGreen™ 488 (Thermo Fisher) and NucBlue™ (Thermo Fisher) counterstaining. Imaging was performed on a Leica DM-8 microscope.

#### sG receptor binding and confocal microscopy

A549 cells grown on Lab-Tek™ II CC2™ slides (Thermo Fisher) in FluoroBrite™ DMEM (Thermo Fisher) were treated with heparinase I/III, as described above, chilled on ice for 10 min, and incubated with 150 µg/mL rsG (WT or CX₃C mutant) or BSA in FluoroBrite™ DMEM for 30 min at 4 °C. After being washed, the cells were fixed in 4% PFA, permeabilized with 0.5% saponin in PBS, and blocked with 5% horse-serum in PBS overnight. The slides were incubated with rabbit anti-RSV G (1:250; Sino Biological) and mouse anti-CX_3_CR1 (1:500; Abcam; ab167571), followed by incubation with Alexa Fluor 594–conjugated goat anti-mouse and Alexa Fluor 488–conjugated goat anti-rabbit antibodies (Thermo Fisher, 0.2 µg/mL). Nuclei and F-actin were counterstained with ActinRed™ 555 (Thermo Fisher) and NucBlue™ (Thermo Fisher). Images were acquired on a Leica TCS SP5 confocal microscope.

#### Pull-down assay

His-tag Dynabeads™ (Cat# 10103D; Thermo Fisher Scientific) were equilibrated and subsequently incubated with 150 µg/mL recombinant BSA (negative control), wild-type soluble RSV G protein (rsG WT), or CX3C motif-deficient sG (rsG CX₃C Mut) for 1 h at 4 °C with rotation. To improve assay specificity, lysates from A549 cells (for CX_3_CR1 detection) or THP-1 cells (for TLR2 detection) were precleared by incubation with uncoupled beads, followed by magnetic separation. Precleared lysates (500 µg total protein), prepared in M-PER Mammalian Protein Extraction Reagent (Thermo Fisher Scientific) supplemented with protease inhibitors, were then incubated with bait-loaded beads for 2 h at 4 °C with rotation. Following incubation, the beads were washed five times with ice-cold lysis buffer to remove unbound proteins. The bound proteins were eluted by resuspending the beads in 1× Pierce™ Lane Marker Reducing Sample Buffer (Cat# 39000; Thermo Fisher Scientific) and boiling for 5 min, which simultaneously denatured the proteins and released them from the beads. Eluates were separated by SDS‒PAGE and analysed by Western blot using antibodies specific for the 6 × His tag (to confirm bait protein), CX_3_CR1 or TLR2, followed by HRP-conjugated secondary antibodies and chemiluminescent detection as described above.

#### Membrane-associated sG detection

A549 cells in 24-well plates were treated with heparinase I/III, as described previously, chilled on ice for 10 min, and incubated with 150 µg/mL rsG (WT or CX₃C mutant) or BSA for 30 min at 4 °C on ice. After washing, the membrane and cytosolic fractions were separated via the Mem-PER™ Plus Membrane Protein Extraction Kit (89842; Thermo Fisher). Fractions were resolved by SDS-PAGE, transferred to a PVDF membrane by western blotting and probed with anti-6×His (1:1000) and anti-ATP1A1 (1:50,000) as described above.

#### Effect of sG treatment on viral replication

A549 cells in 24-well plates were infected with RSV-A-0594-eGFP (MOI of 1) and then treated with rBSA or rsG (WT/CX₃C mutant) at 15, 50 or 150 nM. The supernatants were collected at 0, 24, 48 and 72 hpi and subjected directly to TCID₅₀ titration on HEp-2 cells; the GFP fluorescence of the sample wells was measured via a Tecan Spark reader.

#### THP-1 blue NF-κB activation assay

THP-1 blue NF-κB cells (InvivoGen) were seeded in 96-well plates and treated with 50 or 150 nM rBSA, rsG WT, or the rsG CX₃C mutant, with or without 200 nM C29 (MedChemExpress HY-100461) or 50 nM TJ-M2010-5 (MedChemExpress HY-139397) for 1 h. After 18 h, SEAP activity was measured by transferring the supernatant to Quanti-Blue™ reagent (InvivoGen) and reading the absorbance at 650 nm after 2 h.

#### Cytokine profiling

A549 cells were infected (MOI of 1) or treated with 150 nM rBSA, rsG WT, rsG CX₃C mutant, or 10 µg/mL LTA for 24 h. Supernatants were analysed via custom Procartaplex™ multiplex kits (Thermo Fisher) for the following analytes: FGF-2, fractalkine (CX_3_CL1), G-CSF, GM-CSF, IFN-α, IL-1α, IL-1β, IL-6, IL-8 (CXCL8), IL-33, IP-10 (CXCL10), MCP-1 (CCL2), MIP-1α (CCL3), MIP-1β (CCL4), RANTES (CCL5), TNF-α, TRAIL, TSLP, VEGF-A, IFN-β and LAP. After incubation with capture beads, the samples were washed, incubated with a biotinylated detection antibody and streptavidin-PE, washed, and resuspended in sheath fluid. The beads were analysed on a Luminex™ 200 system, and the concentrations were calculated via a five-parameter logistic fit.

#### TUNEL assay

A549 cells in 96-well plates were treated with rBSA, rsG WT or the rsG CX₃C mutant (50/150 nM) or infected with RSV-A-0594 (MOI of 1). After 24 h, apoptosis was detected via a One-Step TUNEL In Situ Apoptosis Kit (Elabscience E-CK-A321) according to the manufacturer’s instructions, which included a DNase I (200 U/mL) positive control. The cells were fixed with 4% PFA, permeabilized with 0.1% Triton-X, incubated with the TUNEL reaction mixture, counterstained with DAPI, and imaged with a Leica DM8 microscope.

#### Caspase-3/7 activity assay

A549 cells were seeded in black-walled, clear-bottom 96-well plates and allowed to adhere overnight. For the inhibitor treatments, the culture medium was removed and replaced with fresh medium containing either 200 nM C29 (TLR2 inhibitor; MedChemExpress, Cat# HY-100461) or 50 nM TJ-M2010-5 (MyD88 inhibitor; MedChemExpress, Cat# HY-139397) 30 min prior to the addition of the recombinant proteins. After this preincubation, the cells were treated with 150 nM rBSA, rsG WT or the rsG CX₃C mutant for 6 h in the presence of the respective inhibitor or left untreated. Camptothecin (10 µM) and camptothecin plus Ac-DEVD-CHO (50 µM) served as positive and negative controls, respectively. Caspase-3/7 activity was quantified via the Cell Metre™ Caspase-3/7 Activity Apoptosis Assay Kit (AAT Bioquest, Cat# 22796) according to the manufacturer’s protocol. After 1 h of incubation with the FAM-DEVD-FMK substrate at 37 °C in the dark, fluorescence was measured at Ex/Em = 480/520 nm via a microplate reader.

#### Caspase-1 activity assay

A549 cells were seeded in black-walled, clear-bottom 96-well plates and allowed to adhere overnight. For inhibitor treatments, the culture medium was removed and replaced with fresh medium containing 200 nM C29 (TLR2 inhibitor; MedChemExpress, Cat# HY-100461), 5 µM MCC950 (NLRP3 inhibitor; MedChemExpress, Cat# HY-12815), or 50 nM TJ-M2010-5 (MyD88 inhibitor; MedChemExpress, Cat# HY-139397) 30 min prior to the addition of recombinant proteins or RSV infection. After this preincubation, the cells were treated with 150 nM rBSA, rsG WT or the rsG CX₃C mutant or infected with RSV-A-0594 (MOI of 1) in the presence of the respective inhibitor(s) or left untreated. The treatment was maintained for 24 h. As an assay control, cells were treated with LPS (1 µg/mL) plus nigericin (10 µM) as a positive control for inflammasome activation, and Ac-YVAD-CHO (50 µM) was included as a negative control. Caspase-1 activity was measured via the Caspase-Glo® 1 Inflammasome Assay (Promega, Cat# G9951) according to the manufacturer’s protocol: 100 µL of Caspase-Glo® 1 reagent was added directly to each well, the plates were incubated for 1 h at room temperature in the dark, and luminescence was measured via a Tecan Spark microplate reader.

#### GSDMD pore formation assay

A549 cells in 24-well plates were treated with 150 nM rBSA, sG WT or sG CX₃C mutant or left untreated, with or without RSV-A-0594 infection (MOI of 1). At 24 and 48 h, the cells were lysed (M-PER; Thermo Fisher), and 10 µg of lysate was analysed via Western blotting for GSDMD (full-length ~53 kDa, cleaved ~35 kDa; MA5-44666) and cyclophilin B (PA1-027A). The lysate of THP-1 cells treated with PMA and LPS served as a positive control.

RT‒qPCR Total RNA from A549 cells (24-well plates) treated with 150 nM rBSA, 50/150 nM rsG (WT/CX₃C mutant), 10 µg/mL LTA, or RSV-A-0594 (MOI of 1) was isolated at 6, 12 and 24 h posttreatment via a Monarch® Spin RNA Isolation Kit (NEB T2110). RT‒qPCR was performed with a Luna® Universal One-Step RT‒qPCR Kit (NEB E3005) and gene-specific primers. The primers were synthesised based on sequences published by OriGene Technologies (Table 2). The expression levels were normalised to those of GAPDH, PPIA and PGK1 via the 2^−ΔΔCq^ method.

**Table 2:** Primers used for SYBR Green qPCR

Gene	Forward primer	Reverse primer
PPIA	GGCAAATGCTGGACCCAACACA	TGCTGGTCTTGCCATTCCTGGA
GAPDH	GTCTCCTCTGACTTCAACAGCG	ACCACCCTGTTGCTGTAGCCAA
PGK1	CCGCTTTCATGTGGAGGAAGAAG	CTCTGTGAGCAGTGCCAAAAGC
NOX1	GGTTTTACCGCTCCCAGCAGAA	CTTCCATGCTGAAGCCACGCTT
NOX2	CTCTGAACTTGGAGACAGGCAAA	CACAGCGTGATGACAACTCCAG
iNOS	GCTCTACACCTCCAATGTGACC	CTGCCGAGATTTGAGCCTCATG
IL-1β	CCACAGACCTTCCAGGAGAATG	GTGCAGTTCAGTGATCGTACAGG
NLRP3	GGACTGAAGCACCTGTTGTGCA	TCCTGAGTCTCCCAAGGCATTC

#### Real-time-Glo™ MT cell viability assay

A549 cells were seeded in black-walled, clear-bottom 96-well plates and allowed to adhere overnight. For inhibitor treatments, the culture medium was removed and replaced with fresh medium containing 5 µM MCC950 (NLRP3 inhibitor; MedChemExpress, Cat# HY-12815) or 5 µM necrosulfonamide (gasdermin D inhibitor; MedChemExpress, Cat# HY-100573) 30 min prior to the addition of recombinant proteins or RSV infection. After this preincubation, the cells were treated with 150 nM rBSA, sG WT or sG CX₃C mutant or infected with RSV-A-0594 (MOI of 1) in the presence of the respective inhibitor(s) or left untreated. Real-time-Glo™ reagents (Promega, Cat# G9711) were then added to each well at a 1× working concentration according to the manufacturer’s protocol. The plates were transferred to a Tecan Spark microplate reader maintained at 37 °C and 5% CO₂, and luminescence was recorded every 30 min for up to 72 h to monitor cell viability kinetics in real time.

#### Nitrate/nitrite colorimetric assay

A549 cells in 96-well plates were treated with 150 nM rBSA, 50 or 150 nM rsG (WT or CX₃C mutant), 5 µg/mL LTA, or 1 µg/mL LPS or infected with RSV-A-0594 (MOI of 1). For each condition, parallel wells were treated with or without 200 nM C29. The supernatants were collected at 6 and 24 h post-treatment or infection. Nitrite and nitrate concentrations were quantified via the Cayman Chemical Nitrate/Nitrite Colorimetric Assay Kit (780001): samples were incubated with nitrate reductase and cofactors, followed by incubation with Griess reagents, and the absorbance was measured at 540–550 nm.

#### Measurement of intracellular total ROS

A549 cells were seeded in black-walled, clear-bottom 96-well plates and allowed to adhere overnight. For experimental treatments, cells were exposed to 50 or 150 nM rBSA or rsG (WT or CX₃C mutant), 5 µg/mL LTA, or RSV-A-0594 (MOI of 1). To validate the assay specificity and sensitivity, cells treated with 100 µM hydrogen peroxide (H₂O₂) served as a positive control for ROS induction. For the negative control, the cells were pretreated with 10 mM N-acetylcysteine (NAC) for 1 h, after which the NAC-containing medium was removed, the cells were washed three times with prewarmed PBS, and the medium was replaced with medium containing 100 µM H₂O₂. All the wells were then loaded with Amplite™ ROS Green working solution from the Cell Metre™ Fluorimetric Intracellular Total ROS Activity Assay Kit (AAT Bioquest) and incubated for 1 h at 37 °C and 5% CO₂. After this loading step, the test compounds (including rBSA, rsG WT, the rsG CX₃C mutant, LTA or RSV) or the control compounds were added to the cells, which were subsequently incubated for an additional hour under the same conditions. The intracellular ROS levels were quantified by measuring the fluorescence at Ex/Em = 490/525 nm via a Tecan Spark microplate reader.

#### Peroxynitrite formation assay

A549 cells were seeded in black-walled, clear-bottom 96-well plates and allowed to adhere overnight. For inhibitor treatments, the culture medium was removed and replaced with fresh medium containing 200 nM C29 (TLR2 inhibitor; MedChemExpress), 20 µM DPI (NADPH oxidase inhibitor; Sigma‒Aldrich), or 50 µM MitoTEMPO (mitochondrion-targeted ROS scavenger; Sigma‒Aldrich) 30 min prior to the addition of recombinant proteins or RSV infection. After this preincubation, the cells were treated with 150 nM rBSA, rsG WT, or the rsG CX₃C mutant or infected with RSV-A-0594 (MOI of 1) in the continued presence of the respective inhibitor(s). SIN-1 (200 µM) was included as a positive control. Immediately after treatment, the Cell Metre™ Fluorometric Intracellular Peroxynitrite Assay Kit (AAT Bioquest) detection reagent was added according to the manufacturer’s instructions. The cells were then incubated with the detection reagent and the respective treatments for 12 h in a Tecan Spark microplate reader maintained at 37 °C and 5% CO₂, with fluorescence (Ex/Em = 490/525 nm) recorded every hour. The maximum fluorescence values for each well were determined.

#### Potassium ion efflux assay

A549 cells were seeded in black-walled, clear-bottom 96-well plates and allowed to adhere overnight. The cells were treated with 150 nM rBSA, rsG WT, or the rsG CX₃C mutant or infected with RSV-A-0594 (MOI of 1). Valinomycin (50 µM) was included as a positive control for potassium efflux. At 0, 24 and 48 h post-treatment or infection, the intracellular potassium levels were assessed via the cell-permeant potassium indicator PBFI-AM (Ion Biosciences, Cat# 3031B). For dye loading, the cells were incubated with PBFI-AM in the presence of PowerLoad™ Concentrate (Thermo Fisher) according to the manufacturer’s instructions, which enhances dye solubilisation and cellular uptake. Briefly, the cells were incubated with the dye-loading solution for 60 min at 37 °C in the dark and then washed twice with assay buffer to remove excess dye. The fluorescence was measured at Ex/Em = 380/505 nm, corresponding to the K⁺-free form of PBFI. An increase in fluorescence at this wavelength indicates potassium efflux from the cells. All measurements were performed using a Tecan Spark microplate reader.

#### JC-1 mitochondrial membrane potential assay

A549 cells were seeded in black-walled, clear-bottom 96-well plates and allowed to adhere overnight. The cells were treated with 150 nM rBSA, rsG WT, the rsG CX₃C mutant, or 10 µg/mL LTA or left untreated and incubated for 24 h at 37 °C with 5% CO₂. For the positive control, the cells were treated with 50 µM CCCP directly before the assay according to the manufacturer’s protocol (MedChemExpress, HY-K0601). After treatment, the cells were washed with PBS and incubated with JC-1 working solution for 20 min at 37 °C in the dark, following the manufacturer’s instructions. The cells were subsequently washed twice with assay buffer. Fluorescence was measured via a Tecan Spark microplate reader at Ex/Em = 525/590 nm (JC-1 aggregates, red) and Ex/Em = 490/530 nm (JC-1 monomers, green). The ratio of red to green fluorescence was calculated for each well as an indicator of the mitochondrial membrane potential.

#### LDH release cytotoxicity assay

A549, HEp-2, HULEC-5a, THP-1 and U937 cells in 48-well plates were infected with RSV-A-0594-eGFP (MOI of 1), treated with 150 nM rBSA, sG WT, or sG CX₃C mutant, or left untreated. Additionally, parallel wells were incubated with or without MCC950 (At 0, 24, 48 and 72 h). The supernatants were collected, mixed 1:1 with LDH Storage Buffer (200 mM Tris-HCl, pH 7.3, 10% glycerol, and 1% BSA), and stored at −20 °C. LDH release was quantified via the Cytotoxicity LDH Assay Kit (MedChemExpress HY-K1090): samples were incubated with working solution, stop solution was added, and the absorbance was measured at 490 nm. The values were normalised to those of mock-infected controls.

#### Quantification and statistical analysis

Statistical analyses were performed via GraphPad Prism 10.0.3. The data are presented as the means ± SDs. The specific statistical tests used and significance levels are indicated in the figure legends.

## Supplementary information

Supplementary material

## Data Availability

The datasets used and/or analysed during the current study are available from the corresponding author upon reasonable request.
