# Supplementary material for: The soluble G protein of respiratory syncytial virus promotes viral dissemination via TLR2-mediated NLRP3 priming and pyroptosis
Source: Npj Viruses. 2026 Jan 27;4:6. doi: 10.1038/s44298-026-00172-x (PMC12847760; doi:10.1038/s44298-026-00172-x)
Supplement: Supplementary file 1 — Supplementary material [file 44298_2026_172_MOESM1_ESM.pdf]

Supplementary Figure 1:

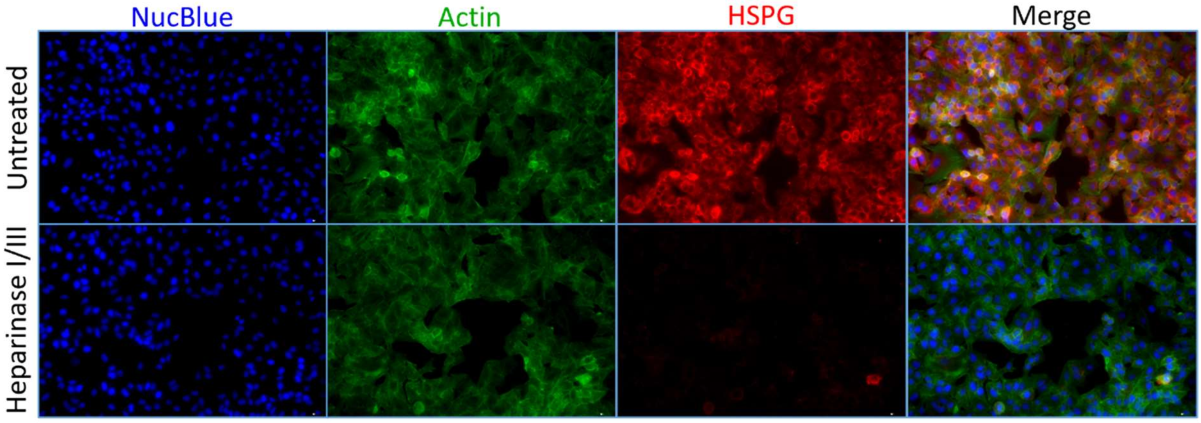

Supplementary Figure 1. Immunofluorescence staining of heparan sulfate proteoglycans (HSPGs), nuclei, and F-actin in A549 A549 cells subjected to heparinase I/III digestion or mock treatment. Immunofluorescence staining of the HSPGs was performed with the F58-10E4 clone (red), with the nuclei counterstained with NucBlue (blue) and F-actin labelled with ActinGreen (green). Representative fields were imaged via a Leica DM-8 fluorescence microscope.

Supplementary Figure 2:

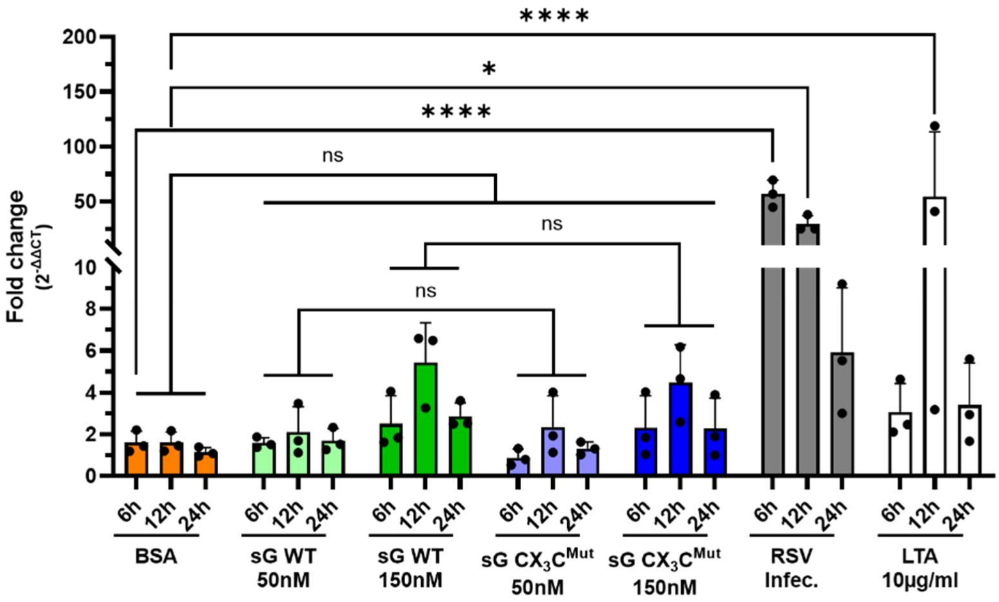

Supplementary Figure 2. RT–qPCR quantification of IL-1β mRNA in A549 cells after sG treatment and RSV infection. Total RNA was isolated from A549 cells treated with rsG (WT or CX_3C mutant), BSA, or LTA or infected with RSV-A-0594. IL-1β transcript abundance was measured via SYBR Green RT–qPCR at the indicated time points via gene-specific primers (see Table 2 in Methods). The data were normalized to that of GAPDH and are presented as the fold change relative to that of the untreated controls, which was calculated via the $2^{-\Delta\Delta Ct}$ method.

Supplementary Figure 3:

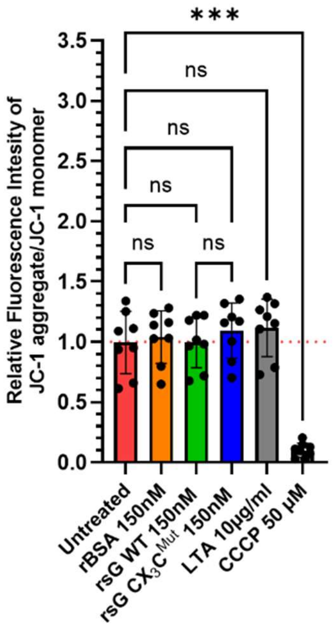

Supplementary Figure 3. To assess the mitochondrial membrane potential (ΔΨm) in A549 cells via the JC-1 fluorescent dye assay, A549 cells were treated with rsG (WT or CX_3C mutant), BSA, LTA, or CCCP (positive control) for 24 h. The cells were incubated with JC-1 working solution, and fluorescence was measured at Ex/Em 525/590 nm (aggregates, red) and 490/530 nm (monomers, green) wavelengths via a Tecan Spark microplate reader. The ratio of aggregated to monomeric fluorescence reflects the ΔΨm status.

Supplementary Figure 4:

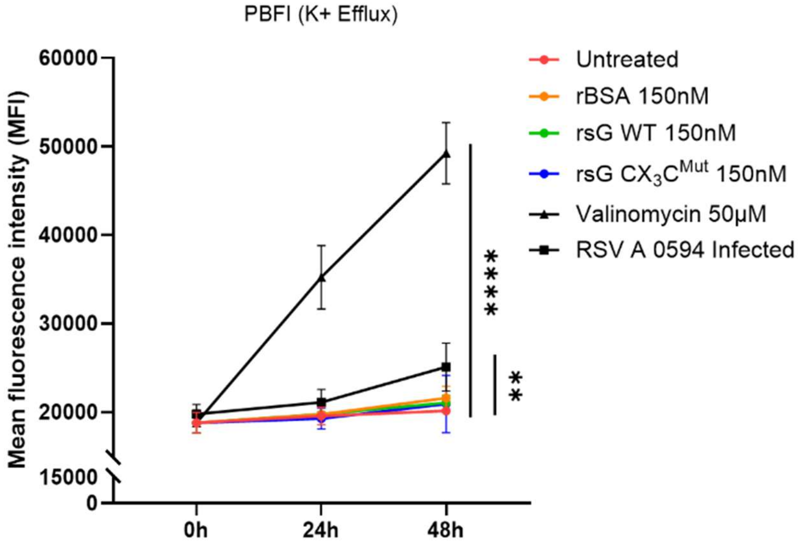

Supplementary Figure 4. Measurement of potassium efflux in A549 cells by the PBFI-AM fluorescent indicator A549 cells were exposed to rsG (WT or CX_3C mutant) or BSA or infected with RSV-A-0594. Valinomycin served as a positive control for K^+ efflux. Intracellular K^+ levels were assessed at the indicated time points posttreatment via PBFI-AM and PowerLoad concentrate per the manufacturer's instructions. The fluorescence was recorded at Ex/Em = 380/505 nm (K^+-free form) via a Tecan Spark microplate reader.
